# Development of an infection via sugar meal (IVSM) technique to infect mosquitoes (Diptera: Culicidae) with arthropod-borne viruses

**DOI:** 10.1371/journal.pntd.0014715

**Published:** 2026-11-02

**Authors:** Ivan Drahun, Amanda J. M. Williams, Larissa R. Barelli, Larissa Krokovsky, Fiona F. Hunter

**Affiliations:** 1 Department of Biological Sciences and Centre for Vector-Borne Diseases, Brock University, St. Catharines, Ontario, Canada; 2 Faculty of Mathematics & Science and the Office of the Vice-President of Research, Brock University, St. Catharines, Ontario, Canada; 3 Research and Development Division, Entomogen Inc., St. Catharines, Ontario, Canada; Connecticut Agricultural Experiment Station, UNITED STATES OF AMERICA

## Abstract

The global expansion of mosquito vectors is increasing human exposure to arboviruses, highlighting the need for rapid and practical approaches to assess vector competence. Standard artificial laboratory infection methods rely on infectious blood meals or intrathoracic inoculation, both of which can be resource-intensive and limit the capacity to conduct timely experiments, particularly in high-containment facilities. We developed an infection via sugar meal (IVSM) technique to determine whether mosquitoes can acquire and transmit arboviruses by ingesting virus-spiked sucrose. Using IVSM, we tested the vector competence of *Aedes albopictus* and *Culex quinquefasciatus* for West Nile virus (WNV), Mayaro virus (MAYV), and Zika virus (ZIKV) following 1-hour or 24-hour exposures. Viral RNA copy numbers were quantified in the body, legs/wings, and saliva at 7 and 10 days post-infection. Both species supported WNV infection and dissemination, with the virus detected in saliva. *Aedes albopictus* exhibited high MAYV infection and dissemination rates, whereas *Cx. quinquefasciatus* was largely refractory. ZIKV infection and dissemination were limited in both species and did not progress to saliva. High feeding success, low vector mortality, and reproducible outcomes across trials demonstrate that IVSM is an accessible and practical alternative to blood-based infection models and provides a framework for investigating vector competence under conditions that utilize natural feeding behavior.

## Author summary

Mosquito-borne viruses are increasing in prevalence globally, partly due to the expansion of mosquito populations into new geographic regions. To investigate the mechanisms by which these viruses move through the mosquito body and become transmissible, researchers commonly administer infectious blood meals or inject the virus directly into the vector. However, these methods can be technically demanding, require specialized equipment or animal-derived products, or may not represent natural mosquito feeding behaviors. This study introduces and evaluates a novel technique, infection via sugar meal (IVSM), in which mosquitoes are exposed to a sugar solution with infectious virus. Sugar feeding is an innate behavior that provides mosquitoes with energy. The technique was tested on two widely distributed mosquito species, *Aedes albopictus* and *Culex quinquefasciatus*, using three medically significant arboviruses. The progression of viral infection was quantified in the bodies, legs and wings, and saliva of the mosquitoes. Our results demonstrate that virus acquired via sucrose ingestion can establish infection and disseminate throughout the mosquito, eventually reaching the saliva. The IVSM approach resulted in high feeding success, low mortality, and reproducible outcomes, offering a practical alternative to blood- and injection-based infection methods, highlighting its utility for vector competence research.

## Introduction

Arthropod-borne viruses (arboviruses) are among the most significant emerging and re-emerging pathogens, transmitted to humans by mosquitoes, ticks, and sandflies [1]. Arboviruses transmitted by mosquitoes (Diptera: Culicidae) such as dengue (DENV), chikungunya (CHIKV), Mayaro (MAYV), Usutu (USUV), West Nile (WNV), yellow fever (YFV), and Zika (ZIKV) are increasing globally with respect to their emergence, incidence, and distribution [1–3]. Driven by climate change and sociodemographic shifts such as globalization, urbanization, and changes in land use patterns, mosquito vectors and their associated pathogens are rapidly expanding their ranges [3–5]. These range expansions also increase the likelihood that non-native pathogens and arthropod vectors will come into contact, potentially creating new pathogen-vector systems [6].

WNV (genus *Orthoflavivirus*; family *Flaviviridae*) was first identified in 1937 in a woman from the West Nile district of Uganda [7]. It was introduced into North America in 1999 and has since spread rapidly, becoming endemic within a few years [8–10]. While most human infections are asymptomatic, febrile illness appears in 20% of infected people. In ∼1% of patients, the virus can invade the central nervous system, leading to encephalitis, acute flaccid paralysis, seizures, and long-term neurological complications [11–13]. WNV has become the leading cause of mosquito-borne neuroinvasive disease in the United States and Canada, resulting in thousands of confirmed cases each year [14,15]. Its ecological success is due in part to the adaptability of *Culex* mosquito vectors and broad avian reservoir host range [8]. ZIKV (genus *Orthoflavivirus*; family *Flaviviridae*) was historically restricted to Africa and Asia. It caused sporadic outbreaks in Micronesia (2007), French Polynesia (2013), and several Pacific islands (2015), gaining global attention during the 2015–2016 epidemics in the Americas [16–18]. ZIKV infections in Brazil prompted substantial public health challenges, primarily due to the increased associations with microcephaly and other neurodevelopment disorders in newborns of infected mothers, in addition to febrile illness and Guillain–Barré syndrome [16,19,20]. MAYV (genus *Alphavirus*; family *Togaviridae*) has historically been confined to sylvatic cycles in South America, involving *Haemagogus* mosquitoes and non-human primates [21]. However, recent growth in urban *Aedes* mosquito populations raises concerns about an ecological shift similar to that observed with CHIKV, which facilitated rapid global spread [5]. Although currently underreported, MAYV has the potential to cause debilitating arthralgia and prolonged morbidity, posing a growing threat to regions already burdened by DENV, ZIKV, and CHIKV [5]. Mosquitoes belonging to the genera *Aedes* and *Culex* are of major public health concern due to their ability to transmit several arboviruses. *Aedes aegypti* (Linnaeus) and *Aedes albopictus* (Skuse) are vectors of CHIKV, DENV, MAYV, YFV, and ZIKV, whereas *Culex pipiens* (Linnaeus) and *Culex quinquefasciatus* (Say) can transmit USUV and WNV [3,22–24]. However, these viruses are not exclusive to specific genera; *Aedes* mosquitoes, such as *Aedes vexans* (Meigen) and *Ae. albopictus* can also transmit WNV [25,26], while ZIKV replication has been observed in *Cx. quinquefasciatus* [27–29].

Vector competence refers to the physiological ability of a female mosquito to acquire, sustain, amplify, and eventually transmit the virus [30–32]. For a female mosquito to become infected, it must first acquire a virus during a blood meal from an infected host. The virus must then infect and replicate within the midgut epithelial cells, overcoming the midgut infection barrier (MIB), midgut escape barrier (MEB), salivary gland infection barrier (SGIB), and salivary gland escape barrier (SGEB) [1,31,33,34]. Only after the salivary glands are infected, and the virus is secreted into the saliva, can transmission occur during subsequent blood feeding. The effectiveness of these tissue barriers is highly variable and influenced by viral genotype, mosquito genetics, extrinsic and intrinsic factors, temperature, and microbiome composition [1,30]. The concept of vector competence, therefore, serves not only as a mechanistic explanation for why certain viruses spread in specific contexts but also as a predictive framework for anticipating outbreaks and designing vector control interventions.

Vector competence experiments have traditionally relied on three approaches: blood-feeding on an infectious host animal, feeding on an infectious blood meal using a “blood sausage” or membrane feeder, and intrathoracic inoculation. Infection achieved by feeding on an infected vertebrate host has financial limitations (purchase and maintenance of animals), along with rigorous ethical considerations, which make artificial feeders a more accessible method [35]. Artificial blood feeding through membrane feeding systems mimics the natural route of infection but is affected by factors such as blood composition, viral stability/infectivity, and the absence of initiating host cues, leading to variability in feeding and infection rates. Conversely, intrathoracic injections bypass the MIB and MEB, crucial midgut tissue barriers, thereby overestimating transmission capabilities while also requiring fine technical skill, specialized equipment, and cuticle puncture that can result in a heightened immune response and even death of the mosquito due to needle trauma [36,37]. The limited accessibility or scalability of these methods, particularly in high-containment laboratories, underscores the need for alternative approaches that balance biological relevance with logistical feasibility and practicality.

While only anautogenous female mosquitoes require blood to complete oogenesis, all culicids rely on plant-derived sugar sources (e.g., floral and extrafloral nectar, honeydew, tree sap, rotting or damaged fruit, etc.) for energy and survival [38]. Sugar feeding offers a natural and underexplored opportunity for mosquito research, having already been implemented in several other contexts [39–41]. From a practical standpoint, a method that centers on sugar feeding eliminates the need for vertebrate blood and simplifies logistics by avoiding animal handling and the use of specialized feeding apparatuses. Importantly, using an infectious sugar meal leverages the innate sugar feeding behavior of mosquitoes, ensuring that infection processes, such as midgut invasion and dissemination, remain comparable to natural transmission cycles.

In this study, we developed and implemented an infection via sugar meal (IVSM) technique to expose *Cx. quinquefasciatus* and *Ae. albopictus* mosquitoes to WNV, MAYV, and ZIKV to assess whether this method could successfully result in infected and infectious mosquitoes. We show that virus-spiked sugar meals can lead to disseminated infections and can be detected in mosquito saliva, demonstrating that IVSM is a valuable technique for investigating virus-vector interactions. This accessible, simplified, high-throughput, and effective technique will serve as a critical tool for identifying vectors of emerging pathogens and for informing evidence-based control strategies to safeguard public health.

## Materials and Methods

### Mosquitoes

Two mosquito species, *Aedes albopictus* (strain: ATM-NJ95) and *Culex quinquefasciatus* (strain: JHB, NR-43025), were provided by the Centers for Disease Control and Prevention for distribution by BEI Resources, NIAID, NIH. Larvae were maintained with ground TetraFin fish food. Adult mosquitoes were maintained at 27 ± 2°C and ~75% relative humidity (RH), under a 12:12 hr (light:dark) photoperiod, with a 10% sucrose solution supplied *ad libitum* [23,42]. Weekly blood feedings, consisting of citrated sheep blood (Cedarlane, CL2581-1000C) supplemented with 2 μM adenosine-5’-triphosphate disodium salt hydrate (Alfa Aesar, J61125-09), were done using a Hemotek membrane feeding apparatus (Hemotek Ltd., Blackburn, UK).

### Cells and virus strains

The WNV strain, NY99 (GenBank acc. DQ211652), was obtained from the National Microbiology Laboratory (Winnipeg, MB). The MAYV strain, TRVL 15537 (Accession No. KP842810), was purchased from ATCC (ATCC VR-1863). The two ZIKV strains, PRVABC59 (ATCC VR-1843; GenBank acc. KU501215) and R116265 (ATCC VR-1868; GenBank acc. KX766029), were ordered from BEI Resources.

Virus stocks were propagated in Vero cells CCL-81 under Biosafety Level 3 (BSL-3) conditions at Brock University. Cells were cultured in Dulbecco’s minimal Eagle’s medium (DMEM) (Sigma-Aldrich, D6429), high glucose, supplemented with 10% fetal bovine serum (FBS) (Sigma-Aldrich, F1051) and 2% glutamine-penicillin-streptomycin solution. Cells were incubated at 37°C under 5% CO_2_ conditions. Cells were cultivated in culture flasks to an 80–90% confluent monolayer and infected with a specific virus (WNV, MAYV, or ZIKV) at a multiplicity of infection (MOI) of 0.02. After notable cytopathic effects were observed, the culture media was harvested and centrifuged (5000 g, 30 mins, 4°C), and clarified stock transferred into 0.5 mL aliquots, and stored at -80°C until further use. Viral titers were determined using plaque assays as described in Agbulos et al., (2016) [43].

### Infections via sugar meals (IVSMs) with arboviruses

One- to two-day-old mosquitoes were starved from emergence to promote more sugar meal ingestion during experiments. Clear, round plastic cups (11 cm D × 8 cm H) were fashioned into mosquito experimental containers (Fig 1). A square was cut from the lid, and fine mesh was hot glued to allow cotton balls soaked in 10% sucrose to be placed to maintain adults after infection. A 2.5-inch (6.35 cm) trough was created with hot glue on one side of the container that would house the infectious sugar meal and ensure that it did not migrate throughout the entire container (Fig 1A). A hole (< 1 mm) was created in the center of the wall that the trough was attached to, approximately 1 cm from the bottom. This was used as the entry point of the syringe to deliver and remove the infectious sugar meal. On the other side of the container, a cross incision was carved out with a blade to be used as a means of transferring adult mosquitoes from their fabric cages into the experimental containers using an aspirator. Mosquitoes were transferred into experimental containers and placed in an incubator (27 ± 2°C and ~75% RH) one day prior to infection in the biosafety level 3 (BSL-3) laboratory for acclimation. Once mosquitoes were transferred into the container, the incision was sealed to prevent mosquitoes from escaping.

**Fig 1:**
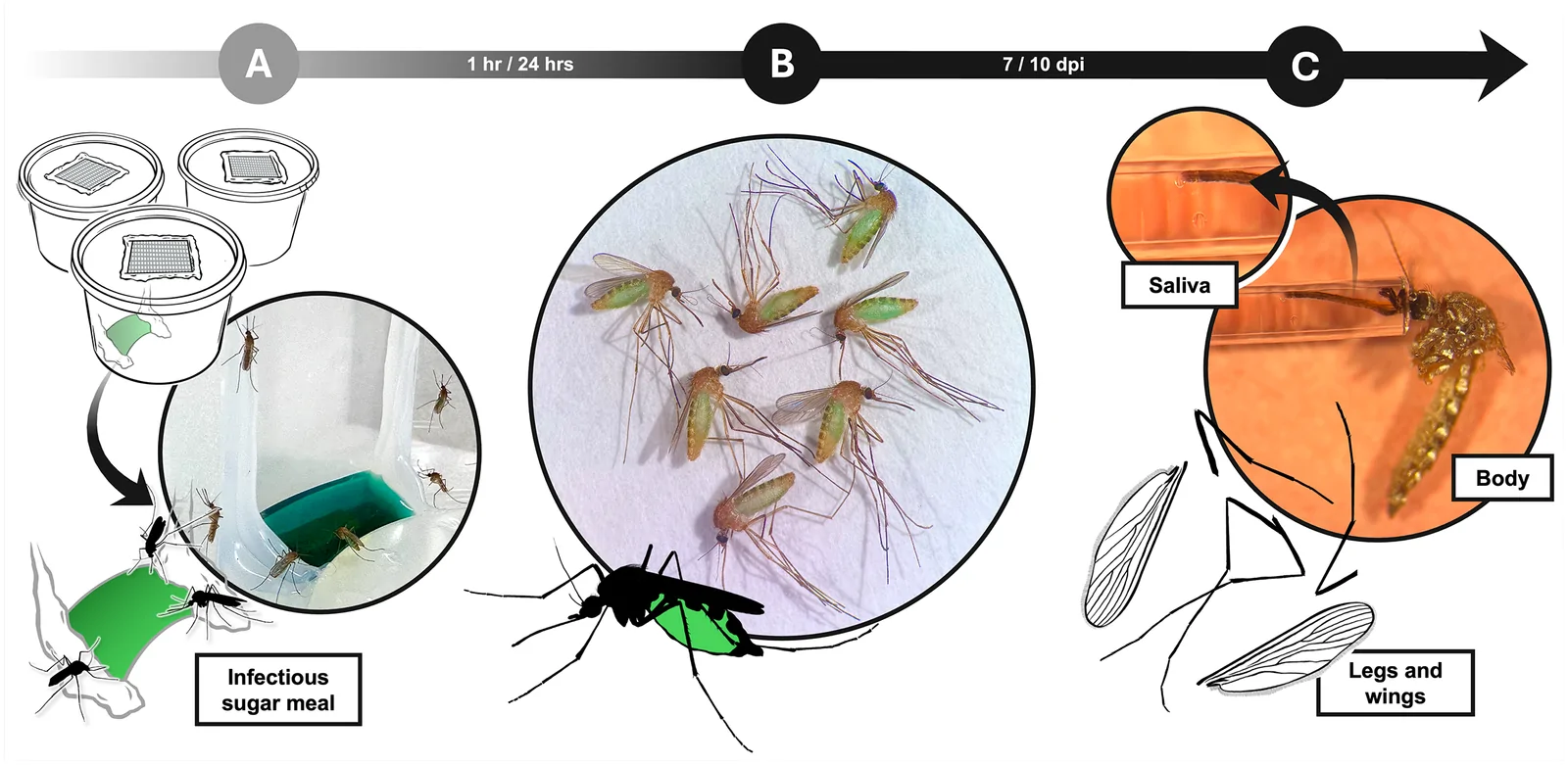
Overview of the IVSM technique for arbovirus infection in mosquitoes. **(A)** Experimental setup showing sealed containers with a constructed trough encompassing an infectious sugar meal prepared with green food dye. Mosquitoes were allowed to feed for either 1 hr or 24 hrs under BSL-3 conditions. **(B)** A visibly green, distended abdomen indicative of successful feeding and ingestion of the infectious sugar meal. Fed mosquitoes were transferred to clean holding containers and supplied with non-infectious 10% sucrose diets to assess vector competence. **(C)** At 7 and 10 dpi, individuals were dissected: legs and wings (LW), bodies (BD), and saliva (SL) were collected for RNA extraction and RT-qPCR to determine viral RNA copy number in infection (BD), dissemination (LW), and transmission (SL) tissues. Images and graphics by Drahun, I.

Infectious sugar meals were a final volume of 1 mL per experimental container and consisted of a 5% sucrose solution diluted with DMEM and a given arbovirus. Sugar meals were prepared with WNV and MAYV in the final concentration of 1.0 × 10^7^ PFU/mL, whereas ZIKV infections were calculated for 1.9 × 10^6^ PFU/mL. Green food coloring dye (Great Value Assorted Food Color) was added (~5 µl of dye per 1 mL of solution) to the final solution and used as a visual marker of successful feeding. Experimental containers were placed into a Sterilite 11.4 L Gasket Box (41 × 28.6 × 17.1 cm) within a biological safety cabinet for the duration of the infection. The air-tight box was used to mitigate disruption to mosquito feeding due to the cabinet airflow while satisfying biosafety requirements of the BSL-3 facility. Feedings were conducted in the afternoon (between the hours of 15:00 and 17:00). Adults were given 1 hr or 24 hrs to feed (~23°C) and then anesthetized using a CO_2_ knockout plate to be sorted. Those with a visibly green abdomen (Fig 1B) were transferred into new experimental containers without a trough, where they were maintained before being dissected on the predetermined days post infection (dpi). Females that were not engorged or took incomplete meals were excluded. Experimental containers containing mosquitoes were housed in metal mesh cages (30 cm L × 30 cm W × 30 cm H, Bioquip Products Inc.) as a secondary containment within an incubator and maintained at conditions of 27 ± 2°C and ~75% RH. Sucrose cotton balls were replaced every 2–3 days.

### Mosquito sample collections

After either 7 or 10 dpi, mosquitoes were sedated using CO_2_ to permit dissection. The legs and wings of each female were removed and collected into a 2 mL microcentrifuge tube for RNA extraction. After ensuring each female was still alive, the proboscis was then inserted into a capillary tube (Drummond Science, 5-000-2005), filled with 1 µl of immersion oil (Fluka Analytical, 10976) to collect the saliva, through forced salivation (FS). Salivation was visually confirmed by the presence of saliva bubbles trapped within the oil (Fig 1C). After ~1 hr of expectoration, females were confirmed to be alive by physical stimulation, and the bodies were collected into a 2 mL microcentrifuge tube, followed by saliva ejection into a 1.5 mL microcentrifuge tube containing 100 µL of DMEM. Equipment was surface decontaminated with 70% ethanol (EtOH) in between handling the legs and wings, bodies, and saliva samples to prevent cross-contamination. Capillary tube plungers were submerged into a 10% bleach solution, followed by 70% EtOH, and wiped dry after each saliva sample was collected. All samples were stored at -80°C until RNA extraction.

### Side-by-side IVSM and blood feeding experiments

To determine the effect of the IVSM technique compared to traditional membrane feeding using blood, female *Ae. albopictus* and *Cx. quinquefasciatus* were collected 2 and 7 days-post-eclosion (dpe) and exposed to either an infectious sugar meal or infectious blood meal. Due to the differences in the timeline for each method, several comparisons were performed. Firstly, mosquitoes collected on day two followed the timeline of the IVSM technique; after eclosion, females were completely devoid of sugar and deprived of water 24 hrs prior to infection, for both IVSM and blood-feeding experiments (categorized as 2 dpe). Secondly, to align with the timing of blood feeding, adult females were maintained on 10% sucrose solution for six days following eclosion and then starved and deprived of water 24 hrs before infection on the seventh day, for both IVSM and blood-feeding experiments (categorized as 7 dpe).

Feeding experiments were performed side-by-side using separate Sterilite containers, one of which had holes created in the lid to allow the cable of the Hemotek FU1 Feeder to be fed through to the power unit. The Hemotek collagen membrane was used for blood feeding experiments; in addition, a pork sausage casing membrane was used to increase feeding in *Ae. albopictus* following poor rates using the standard collagen membrane rubbed on human skin. Immediately before feeding, the collagen membrane was rubbed with bird feathers to transfer avian host odors to the membrane surface, with the aim of enhancing *Cx. quinquefasciatus* blood feeding. A feeder was placed on top of the mesh of the experimental container to allow the females to blood feed. The holes in the lid were sealed with Parafilm to create an airtight barrier. Infectious meals were prepared with WNV final concentration of 1.0 × 10^7^ PFU/mL and 1.0 × 10^5^ PFU/mL. In addition, a side-by-side comparison of blood meal and IVSM ZIKV infections was performed using only *Ae. albopictus*, at a titer of 1.9 × 10^6^ PFU/mL. *Aedes albopictus* was prioritized as it is one of the primary vectors of ZIKV and given that our results showed no transmission and only negligible dissemination in *Cx. quinquefasciatus*. After the 1 hr exposure to the infectious meal, mosquitoes were anesthetized with CO_2_ and sorted into new experimental containers. Dissections were performed at 10 dpi following the previously outlined method. As described earlier, mosquito feedings were conducted between 15:00 and 17:00 for all experiments. Although dawn or dusk may coincide more closely with the natural blood feeding activity of some mosquito species, a standardized feeding window was maintained across all experimental groups to ensure consistency. This should be considered when interpreting overall blood feeding success.

### RNA isolation and RT-qPCR

RNA was extracted from mosquito specimens using RNeasy 96 Kit (Qiagen, 74182), following the manufacturer’s instructions. For the tissue homogenization of the legs and wings, and bodies, 200 µl of DMEM and a sterile stainless-steel bead were added to the tube and lysed with a Tissue Lyser II (Qiagen) at 30 oscillations/second for 30 sec. Total RNA was extracted from 100 µl of the homogenate as per the manufacturer’s instructions, eluted in 70 µl of RNase-free water, and stored at -80°C until use. Direct-to-PCR was performed with saliva samples, as per in-house experimentation demonstrated that this method performed equal to extraction (S1 Table). Saliva samples were thawed at room temperature and thoroughly mixed. 10 µl of sample was added to a 96-well PCR plate and then placed into a thermocycler (Eppendorf AG 22331, Model: 5341) for inactivation at 95°C for 15 mins, followed by a hold at 4°C. This heat inactivation permitted the removal of the sample from the BSL-3 facility for RT-qPCR analysis. Inactivated samples were stored at -80°C until use.

Viral RNA was detected using a one-step RT-qPCR. For each virus, a 12.5 µl reaction was prepared using the QuantiNOVA Probe RT-PCR Kit (Qiagen, 208356) following the manufacturer’s instructions, with 1 µM of each primer, 125 nM probe, and 2.5 µl of template. The sequences of the primers and probes are outlined in Table 1. RT-qPCR reactions were performed using a thermocycler (Bio-Rad Laboratories, CFX Connect) under the following conditions: 45 °C for 10 min, 95 °C for 5 min, followed by 40 cycles of 95 °C for 10 sec and 60 °C for 30 sec. All samples were tested in duplicate, with negative reaction controls (i.e., no template) and positive controls (i.e., standard curve) on each plate. The standard curves for all three viruses were synthesized with *in vitro* transcription using the Invitrogen MEGAscript T7 Transcription Kit (Thermo Fisher Scientific, AMB13345) and quantified using a Nanodrop 2000. RNA concentration was converted into RNA copy number [44]. Samples with cycle quantification (Cq) values ≤ 38.57 for MAYV, ≤ 38.23 for WNV, and ≤ 36.22 for ZIKV were considered positive, if detected in duplicate, and then averaged for RNA copy number.

**Table 1:** Primer and probe sequences used for RT-qPCR detection of MAYV [45], WNV [46], and ZIKV [17].

**MAYV**	Forward	5’ CAC GGA CMT TTT GCC TTC A 3’
	Reverse	5’ GAC TGC CAC CTC TGC TKG AG 3’
	Probe	5’**(HEX)** ACA GAT CAG ACA TGC AGG 3’
**WNV**	Forward	5’ TCA GCG ATC TCT CCA CCA AAG 3’
	Reverse	5’ GGG TCA GCA CGT TTG TCA TTG 3’
	Probe	5’(**FAM)** T GCC CGA CCA TGG GAG AAG CTC 3’
**ZIKV**	Forward	5’ CCG CTG CCC AAC ACA AG 3’
	Reverse	5’ CCA CTA ACG TTC TTT TGC AGA CAT 3’
	Probe	5’**(HEX)** AGC CTA CCT TGA CAA GCA GTC AGA CAC TCA A 3’

### Data analysis

Infection rates (IR = positive bodies/total dissected), dissemination rates (DR = positive legs and wings/positive bodies), and transmission rates (TR = positive saliva/positive legs and wings) were calculated for each virus and each species at different time points. To compare the RNA copy number of positive samples, the non-parametric Mann-Whitney test and the Friedman test were used. Analyzed results were considered significant when the *p* values were < 0.05. All statistical tests were performed with GraphPad Prism 10 software (GraphPad, San Diego, CA, USA).

## Results

Using the IVSM technique, we infected 423 *Ae. albopictus* and 388 *Cx. quinquefasciatus* mosquitoes with WNV and MAYV (titer of sugar meals, 1.0 × 10^7^ PFU/mL) and ZIKV (sugar meal titer 1.9 × 10^6^ PFU/mL), by means of three independent trials for each species, in experimental containers at two exposure times (1 hr and 24 hrs) (Fig 1). Both species showed high feeding rates with all three viruses. *Aedes albopictus* had an overall feeding success of 92.5% (WNV: 91.1%; MAYV: 95.1%; ZIKV: 91.3%), and *Cx. quinquefasciatus* had an overall feeding success of 91.7% (WNV: 92.6%; MAYV: 92.1%; ZIKV: 90.5%). The ability to feed mosquitoes a couple of days after emergence also had a positive effect on their survival with all the viruses, with 95.6% and 96.2% of *Ae. albopictus* surviving until the point of collection after 1 hr and 24 hr feedings, respectively. *Culex quinquefasciatus* showed the same trend after exposure to any of the viruses, with 93.6% and 96.1% of all mosquitoes surviving after 1 hr and 24 hr feedings, respectively. To assess the potential of the IVSM technique, we evaluated the vector competence of both species for each of the three viruses, using specific tissues as measures of infection progression throughout the mosquito body (i.e., body, legs and wings, and saliva). In addition, IVSM was performed alongside blood feeding infections to compare IVSM with the standard infection technique.

### WNV infection, dissemination, and transmission

A total of 118 *Ae. albopictus* females and 141 *Cx. quinquefasciatus* females were analyzed (Table 2). Dissections were performed at 7 and 10 dpi, and the tissues were tested by RT-qPCR to verify the presence of WNV and to quantify the RNA copies (S1 Data). After a 1-hr feeding period, the IR in *Ae. albopictus* were 88.2% (7 dpi) and 96.4% (10 dpi), and the IR in *Cx. quinquefasciatus* were 78.8% (7 dpi) and 97.2% (10 dpi). After a 24-hr feeding period, the IR were 100% (7 and 10 dpi) in *Ae. albopictus*, and 77.8% (7 dpi) and 97.2% (10 dpi) in *Cx. quinquefasciatus*. *Aedes albopictus* showed a high DR after 1-hr (90% at 7 dpi; 100% at 10 dpi) and 24-hr (100% at 7 and 10 dpi) feeding period. At 7 dpi, *Cx. quinquefasciatus* showed a DR of 80.8% (1 hr) and 100% (24 hrs), with 97.1% (1 hr) and 94.3% (24 hrs) observed at 10 dpi. While positive saliva samples were present in all trials for both species, *Cx. quinquefasciatus* showed a TR of 50% (1 hr) and 44.1% (24 hrs) at 10 dpi, whereas *Ae. albopictus* showed a TR of 22.2% (1 hr) and 21.4% (24 hrs) at 10 dpi. The highest TR in *Ae. albopictus* was observed to be 28.6% (8/28 females) at 7 dpi after a 24-hr feeding.

**Table 2:** Positivity and rates of infection, dissemination, and transmission for WNV in *Ae. albopictus* and *Cx. quinquefasciatus* after IVSM.

Species	hr	dpi	BD +	IR	LW +	DR	SL +	TR
*Ae. albopictus*	1	7	30/34	88.2%	27/30	90%	2/27	7.4%
		10	27/28	96.4%	27/27	100%	6/27	22.2%
	24	7	28/28	100%	28/28	100%	8/28	28.6%
		10	28/28	100%	28/28	100%	6/28	21.4%
*Cx. quinquefasciatus*	1	7	26/33	78.8%	21/26	80.8%	3/21	14.3%
		10	35/36	97.2%	34/35	97.1%	17/34	50%
	24	7	28/36	77.8%	28/28	100%	6/28	21.4%
		10	35/36	97.2%	33/35	94.3%	15/33	45.5%

Legend: BD = body; LW = legs and wings; SL = saliva; IR = infection rate; DR = dissemination rate; TR = transmission rate.

WNV infection, dissemination, and transmission showed similar trends in tissue infection progression across both species, and after both feeding periods. Infection progression is the sequential movement of a virus through the vector as determined by infection, dissemination, and transmission parameters. All conditions resulted in significant differences between all tissue types (*p* < 0.0001) in both *Ae. albopictus* and *Cx. quinquefasciatus*.

Pairwise analyses were used to compare the number of RNA copies of WNV/mL in the same tissues between the two species, and between the dpi (Fig 2A, 2B). No pairwise comparisons were done between different tissue types. In *Ae. albopictus*, following a 1-hr feeding, higher BD viral loads were observed at 10 dpi compared to 7 dpi (U = 326, *n*(10dpi)=28, *n*(7dpi)=34, two-tailed *p* = 0.0334). Similarly, in *Cx. quinquefasciatus*, following a 1-hr feeding, higher BD viral loads were observed at 10 dpi compared to 7 dpi (U = 218.5, *n*(10dpi)=36, *n*(7dpi)=33, two-tailed *p* < 0.0001). No significant differences in BD viral load were observed between the two species at either dpi. The LW viral loads were higher at 10 dpi compared to 7 dpi in both *Ae. albopictus* (U = 309.5, *n*(10dpi)=28, *n*(7dpi)=34, two-tailed *p* = 0.0178) and *Cx. quinquefasciatus* (U = 162, *n*(10dpi)=36, *n*(7dpi)=33, two-tailed *p* < 0.0001). The viral load in LW was higher in *Ae. albopictus* at 7 dpi compared to the *Cx. quinquefasciatus* (U = 401, *n*(*Ae*)=34, *n*(*Cx*)=33, two-tailed *p* = 0.0425). At 10 dpi, the SL viral load in *Cx. quinquefasciatus* was higher than at 7 dpi (U = 389, *n*(10dpi)=36, *n*(7dpi)=33, two-tailed *p* = 0.0017).

**Fig 2:**
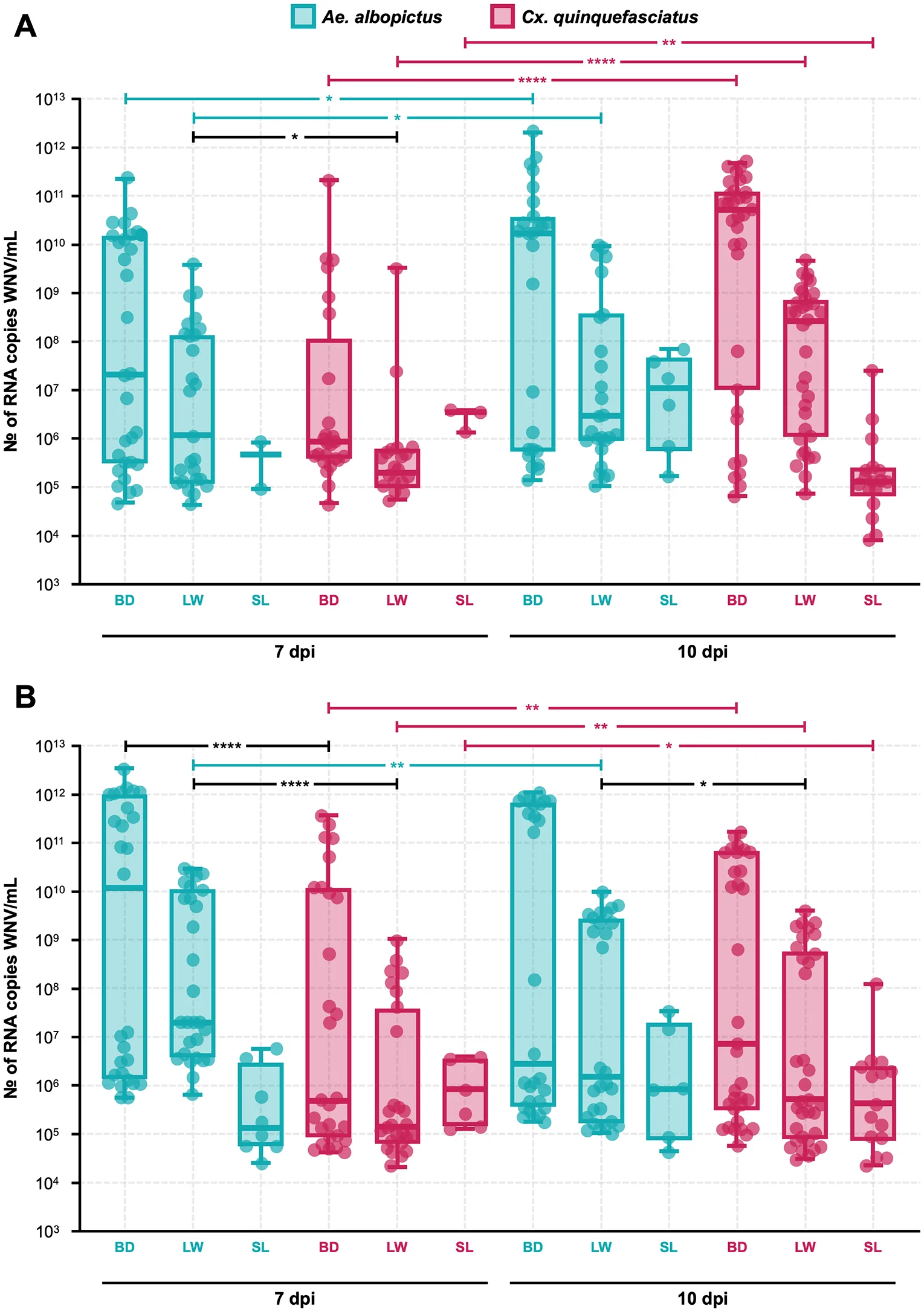
WNV RNA viral loads in *Ae. albopictus* and *Cx. quinquefasciatus* tissues following an IVSM after (A) 1-hour exposure and (B) 24-hour exposure. WNV RNA copy number/mL is shown for body (BD; infection), legs and wings (LW; dissemination), and saliva (SL; transmission) at 7 and 10 dpi for each species. Statistical comparisons used two-tailed Mann-Whitney U tests within the same tissues, testing (i) species differences at each dpi and (ii) 7 versus 10 dpi within the same species; no comparisons were performed between different tissue types. Significance is denoted as * *p* < 0.05, ** *p* < 0.01, *** *p* < 0.001, **** *p* < 0.0001. Non-significant samples were not denoted.

After the 24-hr feeding period, the BD viral load was higher in *Cx. quinquefasciatus* at 10 dpi than at 7 dpi (U = 412.5, *n*(10dpi)=36, *n*(7dpi)=36, two-tailed *p* = 0.0102). A higher BD viral load was observed in *Ae. albopictus* compared to the *Cx. quinquefasciatus* at 7 dpi (U = 121, *n*(*Ae*)=28, *n*(*Cx*)=36, two-tailed *p* < 0.0001). The LW viral load within each species differed with *Ae. albopictus* having a higher load at 7 dpi (U = 225, *n*(10dpi)=28, *n*(7dpi)=28, two-tailed *p* = 0.0056), compared to *Cx. quinquefasciatus* having a higher load at 10 dpi (U = 412, *n*(10dpi)=36, *n*(7dpi)=36, two-tailed *p* = 0.0073). At both 7 dpi, (U = 121, *n*(*Ae*)=28, *n*(*Cx*)=36, two-tailed *p* < 0.0001), and 10 dpi (U = 348, *n*(*Ae*)=28, *n*(*Cx*)=36, two-tailed *p* = 0.0344) *Ae. albopictus* had a higher LW viral load compared to *Cx. quinquefasciatus*. Similar to the 1-hr feeding, only *Cx. quinquefasciatus* had a higher SL viral load at 10 dpi compared to 7 dpi (U = 494, *n*(10dpi)=36, *n*(7dpi)=36, two-tailed *p* = 0.031).

### MAYV infection, dissemination, and transmission

A total of 129 *Ae. albopictus* females and 114 *Cx. quinquefasciatus* females were analyzed (Table 3). Dissections were performed on 7 and 10 dpi, and the tissues were tested by RT-qPCR to verify the presence and quantity of MAYV (S2 Data). After a 1-hr feeding period, the IR in *Ae. albopictus* were 86.2% (7 dpi) and 97.2% (10 dpi), and 17.2% (7 dpi) and 14.3% (10 dpi) in *Cx. quinquefasciatus*. After a 24-hr feeding period, the IR were 96.8% (7 dpi) and 100% (10 dpi) in *Ae. albopictus*, and 10.7% (7 dpi) and 0% (10 dpi) in *Cx. quinquefasciatus*. DR after a 1-hr feeding was 36% (7 dpi) and 94.3% (10 dpi) in *Ae. albopictus*, and 80% (7 dpi) and 50% (10 dpi) in *Cx. quinquefasciatus*. After a 24-hr feeding, DR was 83.3% (7 dpi) and 81.8% (10 dpi) in *Ae. albopictus*, and 33.3% (7 dpi) in *Cx. quinquefasciatus*. *Culex quinquefasciatus* showed no dissemination at 10 dpi. *Aedes albopictus* had overall low TR, with 24.2% (10 dpi) after a 1-hr feeding being the highest. One positive SL sample was seen in *Cx. quinquefasciatus* at 7 dpi after a 24-hr feeding.

**Table 3:** Positivity and rates of infection, dissemination, and transmission for MAYV in *Ae. albopictus* and *Cx. quinquefasciatus* after IVSM.

Species	hr	dpi	BD +	IR	LW +	DR	SL +	TR
*Ae. albopictus*	1	7	25/29	86.2%	9/25	36%	1/9	11.1%
		10	35/36	97.2%	33/35	94.3%	8/33	24.2%
	24	7	30/31	96.8%	25/30	83.3%	1/25	4%
		10	33/33	100%	27/33	81.8%	2/27	7.4%
*Cx. quinquefasciatus*	1	7	5/29	17.2%	4/5	80%	0/5	0%
		10	4/28	14.3%	2/4	50%	0/4	0%
	24	7	3/28	10.7%	2/3	33.3%	1/2	50%
		10	0/29	0%	—	—	—	—

Legend: BD = body; LW = legs and wings; SL = saliva; IP = infection rate; DP = dissemination rate; TP = transmission rate.

MAYV infection, dissemination, and transmission patterns differed between species, with *Ae. albopictus* showing much higher viral loads throughout the tissues than *Cx. quinquefasciatus*. According to the Friedman test, the means were significantly different (*p* < 0.0001) between the three tissue types in *Ae. albopictus* after both 7 and 10 dpi, and after both the 1-hr and 24-hr feeding periods. In *Cx. quinquefasciatus*, however, significant differences were only observed after the 1-hr feeding period (*p* = 0.0214) at 7 dpi.

Pairwise Mann-Whitney analyses were used to compare the number of RNA copies of MAYV/mL in the same tissues between the two species, and across the dpi (Fig 3A, 3B). No pairwise comparisons were made between different tissue types. In *Ae. albopictus*, following a 1-hr feeding, higher BD viral loads were observed at 10 dpi compared to 7 dpi (U = 177, *n*(10dpi)=36, *n*(7dpi)=29, two-tailed *p* < 0.0001). *Aedes albopictus* also had higher BD viral loads than *Cx. quinquefasciatus* at 7 dpi (U = 75, *n*(*Ae*)=29, *n*(*Cx*)=29, two-tailed *p* < 0.0001) and 10 dpi (U = 16, *n*(*Ae*)=36, *n*(*Cx*)=28, two-tailed *p* < 0.0001). The LW viral loads were higher at 10 dpi compared to 7 dpi in *Ae. albopictus* (U = 61, *n*(10dpi)=36, *n*(7dpi)=29, two-tailed *p* < 0.0001). However, the LW viral loads were higher in *Ae. albopictus* than *Cx. quinquefasciatus* at 10 dpi. No significant differences in the SL viral load were observed in *Ae. albopictus* between the two time points. *Aedes albopictus* did show a higher SL viral load at 7 and 10 dpi compared to *Cx. quinquefasciatus*, which had zero positive SL samples.

**Fig 3:**
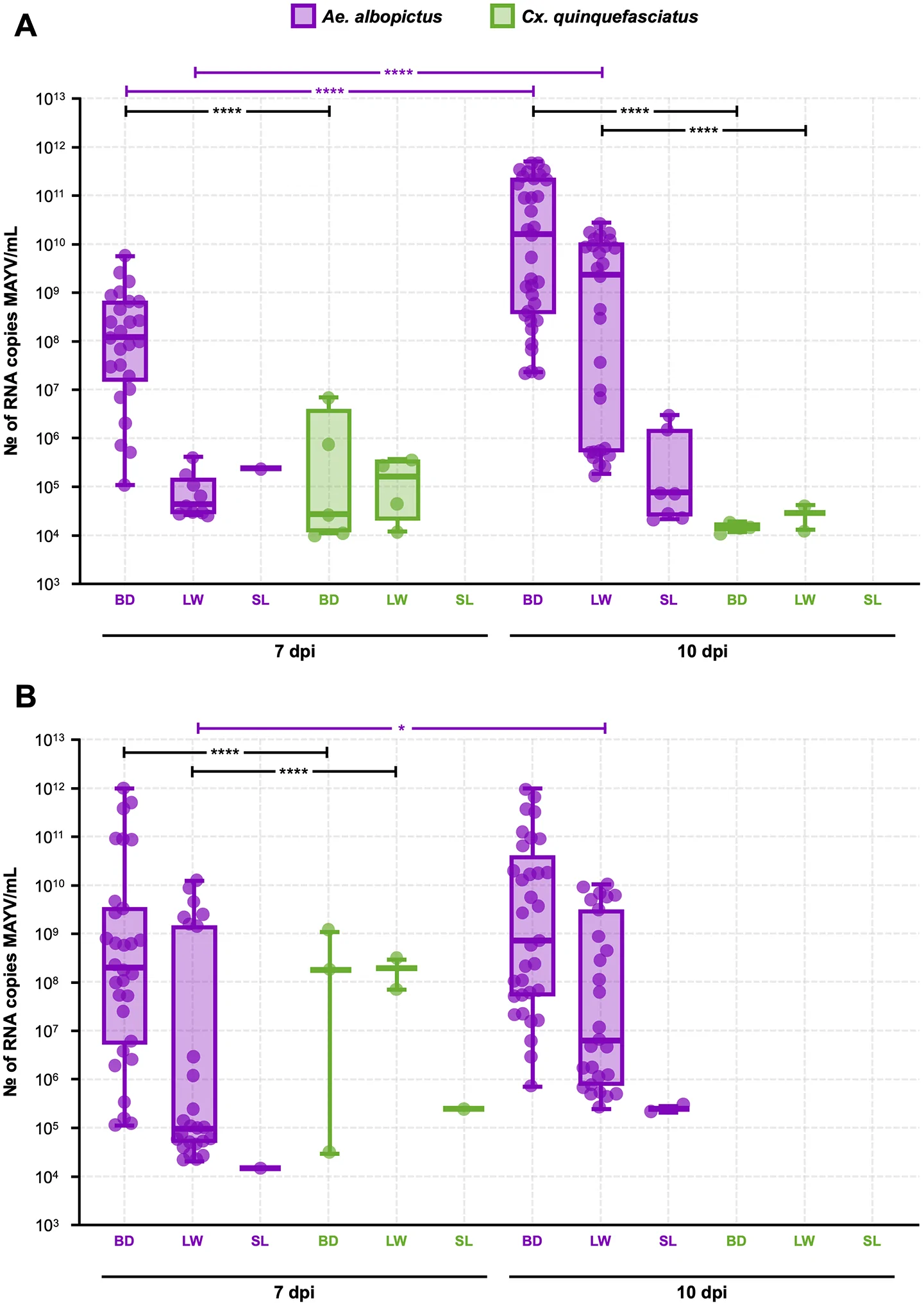
MAYV RNA viral loads in *Ae. albopictus* and *Cx. quinquefasciatus* tissues following an IVSM after (A) 1-hour exposure and (B) 24-hour exposure. MAYV RNA copy number/mL is shown for body (BD; infection), legs and wings (LW; dissemination), and saliva (SL; transmission) at 7 and 10 dpi for each species. Statistical comparisons used two-tailed Mann-Whitney U tests within the same tissues, testing (i) species differences at each dpi and (ii) 7 versus 10 dpi within the same species; no comparisons were performed between different tissue types. Significance is denoted as * *p* < 0.05, ** *p* < 0.01, *** *p* < 0.001, **** *p* < 0.0001. Non-significant samples were not denoted.

After the 24-hr feeding period, the viral load for BD dpi (U = 51.5, *n*(*Ae*)=31, *n*(*Cx*)=28, two-tailed *p* < 0.0001) and LW (U = 126, *n*(*Ae*)=31, *n*(*Cx*)=28, two-tailed *p* < 0.0001) was higher in *Ae. albopictus* than *Cx. quinquefasciatus* at 7 dpi. However, *Cx. quinquefasciatus* showed no positivity at 10 dpi in any of the tissues. No significant differences in the SL viral load were observed between or within the species under any conditions. No further comparisons could be made between MAYV infections between the two species given the low rates of infection, dissemination, and transmission in *Cx. quinquefasciatus*. The only MAYV positive SL sample from *Cx. quinquefasciatus* was obtained following a 24 hr feeding at 7 dpi. This female had a BD infection (1.87 × 10^8^ RNA copies/mL), which was shown to disseminate into the LW (3.02 × 10^8^ RNA copies/mL) and the SL (2.54 × 10^5^ RNA copies/mL).

### ZIKV infection, dissemination, and transmission

A total of 122 *Ae. albopictus* females and 133 *Cx. quinquefasciatus* females were analyzed after infection with ZIKV (Table 4). Dissections were performed at 7 and 10 dpi, and the tissues were tested by RT-qPCR to verify ZIKV presence and quantify viral load (S3 Data). After a 1-hr feeding period, the IR in *Ae. albopictus* were 12.9% (7 dpi) and 7.1% (10 dpi), and 0% (7 dpi) and 2.7% (10 dpi) in *Cx. quinquefasciatus*. After a 24-hr feeding period, the IR were 5.9% (7 dpi) and 13.8% (10 dpi) in *Ae. albopictus*, and 13.3% (7 dpi) and 6.3% (10 dpi) in *Cx. quinquefasciatus*.

**Table 4:** Positivity and rates of infection, dissemination, and transmission for ZIKV in *Ae. albopictus* and *Cx. quinquefasciatus* after IVSM.

Species	hr	dpi	BD +	IR	LW +	DR	SL +	TR
*Ae. albopictus* (ZIKV – PRVABC5)	1	7	4/31	12.9%	2/4	50%	0/2	0%
		10	2/28	7.1%	1/2	50%	0/1	0%
	24	7	2/34	5.9%	1/2	50%	0/1	0%
		10	4/29	13.8%	1/4	25%	0/1	0%
*Cx. quinquefasciatus* (ZIKV – PRVABC5)	1	7	0/34	0%	—	—	—	—
		10	1/37	2.7%	0/1	0%	—	—
	24	7	4/30	13.3%	0/4	0%	—	—
		10	2/32	6.3%	1/2	50%	0/1	0%
*Ae. albopictus* (ZIKV – R116265)	1	7	0/27	0%	—	—	—	—
		10	1/27	3.7%	1/1	100%	0/1	0%

Legend: BD = body; LW = legs and wings; SL = saliva; IP = infection rate; DP = dissemination rate; TP = transmission rate.

*Aedes albopictus* showed a DR of 50% (7 dpi) and 50% (10 dpi) after a 1-hr feeding, and 50% (7 dpi) and 25% (10 dpi) after a 24-hr feeding. *Culex quinquefasciatus* showed no dissemination into the legs and wings for all trials, except for one positive sample (24 hrs) at 10 dpi. Transmission was not observed for ZIKV (PRVABC5) in both species under all conditions. To determine whether the strain of ZIKV contributed to the lower-than-expected infection rates, 54 *Ae. albopictus* females were analyzed after a 1-hr infection with ZIKV (R116265). RT-qPCR with ZIKV (R116265) showed even lower viral presence, with 0% IR at 7 dpi and 3.7% IR at 10 dpi. No dissemination was observed at 7 dpi, and the one positive IR sample at 10 dpi also showed dissemination into the legs and wings. No transmission was observed for ZIKV (R116265) either. It should be noted that although the dissemination rates appear high, the infection rate remained very low in all ZIKV trials. Due to the limited number of ZIKV-positive samples across all parameters considered, statistical analyses could not be performed.

### Side-by-side IVSM and blood feeding experiments

Mosquitoes were dissected at 10 dpi, tested by RT-qPCR to verify the presence of WNV and to quantify RNA copies in their respective tissues to determine infection, dissemination, and transmission rates (S4 Data). A smaller subset of infections was done, to (1) investigate infections with a lower titer of WNV in both species, and (2) determine whether the low vector competence for ZIKV was attributed to the infection method. The feeding rates observed for IVSM were higher with mosquitoes starved from eclosion (2 dpe), being 96.3% and 92.1% in *Ae. albopictus* and *Cx. quinquefasciatus*, respectively. The feeding rates with blood varied between species and dpe (Fig 4), being especially low in *Ae. albopictus* at both 2 dpe (4%) and 7 dpe (10%) with the standard collagen membrane (COL), thereby, requiring *Ae. albopictus* to be fed with a pork intestinal casing membrane (PRK) to achieve feeding rates of 40% (2 dpe) and 55.3% (7 dpe). The handful of *Ae. albopictus* that were fed on a collagen membrane were not carried forward in the statistics beyond feeding rates as comparisons. In *Cx. quinquefasciatus*, blood feeding rates were 55% (2 dpe) and 40% (7 dpe), and were successful with the collagen membrane when rubbed with bird feathers to transfer avian host odors to the membrane surface. We also observed partially fed females following IVSM in both species at 7 dpe, which were excluded from further vector competence studies.

**Fig 4:**
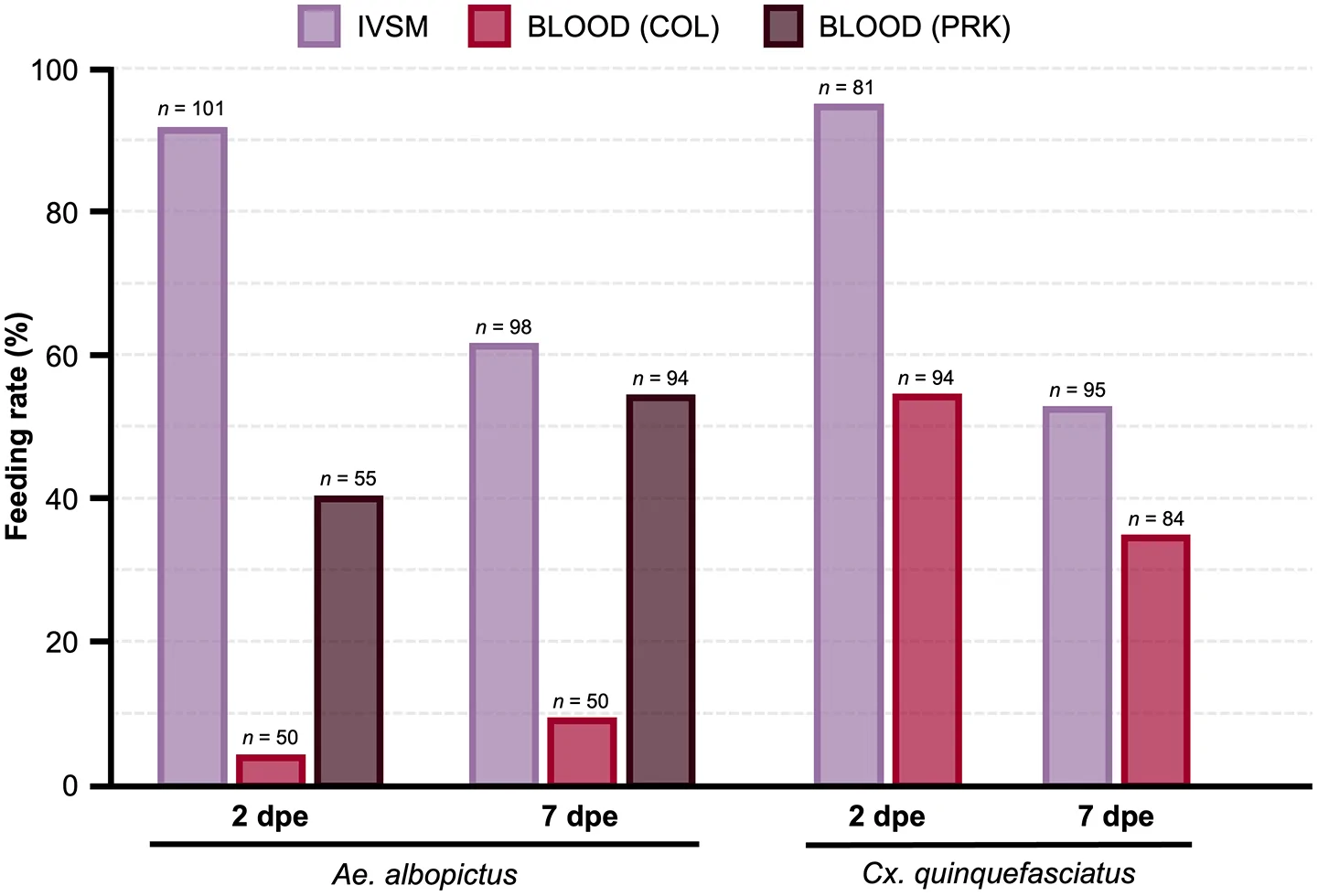
IVSM and blood feeding rates of *Ae. albopictus* and *Cx. quinquefasciatus* during side-by-side infectious sugar- and blood-meal exposures. High feeding rates were achieved with IVSM in both species when starved from pupation and exposed at 2 dpe, whereas feeding rates declined in mosquitoes exposed at 7 dpe. Feeding rates were lower in both species during infectious blood-meal exposure. *Aedes albopictus* required the use of a pork intestinal casing membrane (PRK) as feeding rates with the standard collagen membrane (COL) and human host odors were significantly lower. To promote blood feeding in *Cx. quinquefasciatus*, the collagen membrane was rubbed with bird feathers to transfer avian host odors to the membrane surface.

To determine the rates of infection, dissemination, and transmission in side-by-side experiments, we analyzed 80 *Ae. albopictus* and 73 *Cx. quinquefasciatus* females (Table 5). For WNV (1.0 × 10^7^ PFU/mL), IVSM IR in *Ae. albopictus* were 90.9% (2 dpe) and 89.5% (7 dpe), compared to the blood feeding IR of 79% (2 dpe) and 95% (7 dpe). Similar trends were observed in *Cx. quinquefasciatus*, with IVSM IR of 94.4% (2 dpe) and 89% (7 dpe), and blood feeding IR of 72.2% (2 dpe) and 84.2% (7 dpe). The DR in *Ae. albopictus* were 100% (2 dpe) and 80% (7 dpe) with IVSM, and 94.1% (2 dpe) and 79% (7 dpe) with blood feeding. Conversely, the DR in *Cx. quinquefasciatus* were 100% (2 dpe) and 92.3% (7 dpe) with IVSM, and 100% (2 dpe) and 87.5% (7 dpe) with blood feeding. Transmission was observed in all trials with both IVSM and blood feeding.

**Table 5:** Positivity and rates of infection, dissemination, and transmission for WNV in *Ae. albopictus* and *Cx. quinquefasciatus* after IVSM or blood feeding.

Species	dpe	METHOD	BD +	IR	LW +	DR	SL +	TR
*Ae. albopictus*	2	IVSM	20/22	90.9%	20/20	100%	6/20	30%
		BLOOD	15/19	79.0%	12/15	80%	5/15	41.7%
	7	IVSM	17/19	89.5%	16/17	94.1%	5/16	31.3%
		BLOOD	19/20	95%	15/19	79.0%	4/15	26.7%
*Cx. quinquefasciatus*	2	IVSM	17/18	94.4%	17/17	100%	4/17	23.5%
		BLOOD	13/18	72.2%	12/13	92.3%	4/12	33.3%
	7	IVSM	16/18	89.0%	16/16	100%	7/16	43.8%
		BLOOD	16/19	84.2%	14/16	87.5%	5/14	35.7%

Legend: dpe = days-post-eclosion; BD = body; LW = legs and wings; SL = saliva; IR = infection rate; DR = dissemination rate; TR = transmission rate.

WNV infection, dissemination, and transmission rates did not show significant differences for either of the two species or the two infection methods. Both infection methods and days-post-eclosion (dpe) showed significantly different means across all tissue types (*p* < 0.0001) in both *Ae. albopictus* and *Cx. quinquefasciatus*, according to the Friedman test. Pairwise analyses were used to compare the number of RNA copies of WNV/mL in the same tissues between the two species, and between the two methods of infection (Fig 5A, 5B). No significant differences in BD, LW, or SL viral load were observed between the two species, the two methods of infection, or the two dpe.

**Fig 5:**
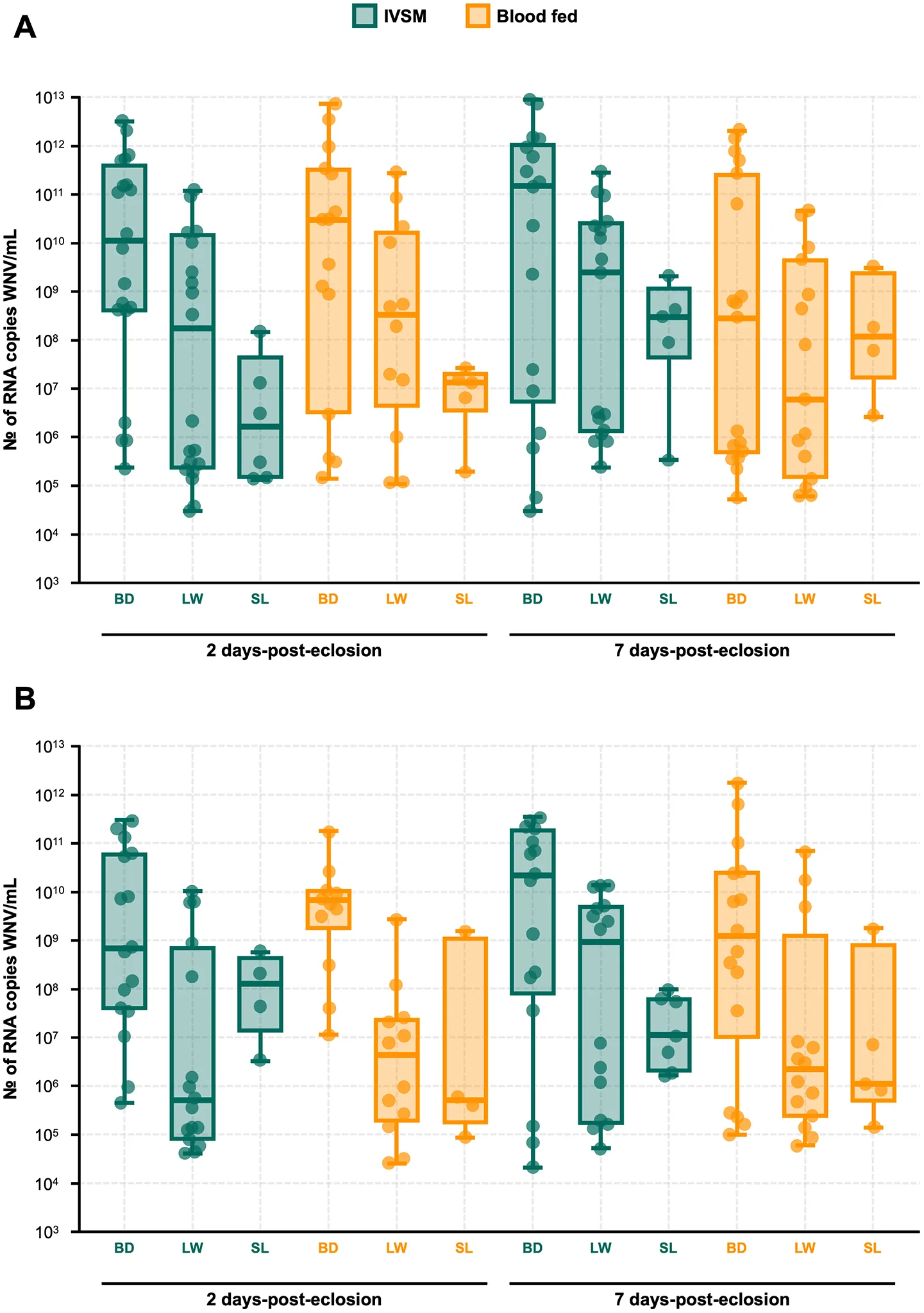
WNV RNA viral loads following an IVSM and a blood feeding at the two day-post-eclosion in (A) *Ae. albopictus* and (B) *Cx. quinquefasciatus* tissues. WNV RNA copy number/mL is shown for body (BD; infection), legs and wings (LW; dissemination), and saliva (SL; transmission) at 2 and 7 dpe for each species. Statistical comparisons used two-tailed Mann-Whitney U tests within the same tissues, testing (i) infection method differences at each dpe and (ii) 2 versus 7 dpe within the same infection method. No significance was observed (*p* > 0.05).

None of the *Cx. quinquefasciatus* infected with WNV at 1.0 × 10^5^ PFU/mL showed infection, dissemination, or transmission between IVSM (0%, 0/20) or blood feeding (0%, 0/21). *Aedes albopictus* showed infection in one IVSM fed mosquito (1.09 × 10^6^ RNA copies/mL) and one blood fed mosquito (2.76 × 10^5^ RNA copies/mL), and dissemination in one IVSM fed mosquito (1.39 × 10^5^ RNA copies/mL) and one blood fed mosquito (1.25 × 10^5^ RNA copies/mL). Each of the positive tissue samples were from individual mosquitoes, therefore, although the IR for IVSM and blood feeding were 4.7% (1/21) and 5% (1/20), respectively; the DR were 0% for both methods. No transmission was observed for either of the two species nor either method following WNV infection 1.0 × 10^5^ PFU/mL.

Side-by-side IVSM and blood meal infections in *Ae. albopictus* with ZIKV at 1.9 × 10^6^ PFU/mL yielded very low IR; in that 5.5% (1/18) and 5% (1/20) bodies were positive via IVSM and blood feeding, respectively. DR was 100% (1/1) via IVSM within the same mosquito but was not present in any of the blood fed mosquitoes. The ZIKV RNA copy numbers/mL for the IVSM IR and DR samples were, 5.29 × 10^7^ RNA copies/mL and 1.07 × 10^6^ RNA copies/mL, respectively. For the blood feeding IR sample, the ZIKV RNA copy number/mL was 1.85 × 10^7^ RNA copies/mL.

## Discussion

This study demonstrates that the infection via the sugar meal (IVSM) technique can successfully establish arboviral infections in *Ae. albopictus* and *Cx. quinquefasciatus*, producing infection, dissemination, and in some cases transmission trends comparable to those achieved through traditional blood meal exposure. By exploiting the natural and ubiquitous mosquito behavior – sugar feeding – this method provides an effective, lower-risk, and scalable alternative for vector competence experiments within high-containment facilities. Laboratories that cannot use animal models typically rely on either artificial blood meals or intrathoracic inoculations, each with substantial drawbacks. Artificial feeding requires vertebrate blood and precise temperature control and often yields inconsistent feeding success among colonies [35]. Given that mosquito blood-feeding behavior is influenced by host preferences and the molecular composition of the meal—which can differentially affect vector physiology—the use of blood can also introduce additional variability in sourcing, storing, and maintaining blood quality [47]. Intrathoracic injection bypasses key tissue barriers, inflating dissemination rates and limiting ecological validity, with the potential to induce cuticular trauma, immune activation, and mortality [36,37]. The IVSM technique resolves these challenges: it retains a route of infection (ingestion) comparable to natural conditions, and eliminates challenges associated with blood sources and artificial blood feeding (temperature control, matching vector to host preference, lot-to-lot variability, availability, and temporal feeding window) [35], by providing a universal and consistent nutritional source (sugar) that bypasses these sources of variability. When considering arboviral studies that require a high-containment facility (e.g., BSL-3), IVSM offers significant practical benefits. Because sugar meals do not contain vertebrate-derived material and can be prepared in sealed, disposable containers, IVSM reduces contamination risks and simplifies decontamination protocols, especially when eliminating the need to decontaminate reusable equipment (e.g., artificial blood feeding reservoirs), thereby reducing the potential for laboratory acquired infections [48,49], decreasing vector stress, and supporting lower-risk biosafety operations. These advantages became evident during side-by-side IVSM and blood feeding experiments, in which a pork intestinal casing membrane was required to achieve acceptable feeding rates in *Ae. albopictus*. The use of this membrane reintroduced animal tissue handling and substantially increased experimental complexity. Once hydrated, the membrane became fragile and challenging to handle. Furthermore, blood permeated the membrane over the span of the 1 hr infection and adhered to the mesh, creating additional decontamination, disposal, and handling requirements throughout the experiment. Despite these additional efforts, only 40% (2 dpe) and 55.3% (7 dpe) of *Ae. albopictus* successfully blood-fed. Which is in keeping with *Cx. quinquefasciatus* feeding rates of 55% (2 dpe) and 40% (7 dpe) via a collagen membrane rubbed with bird feathers. Both species required a feeding cue to promote blood feeding, thereby introducing various new measures for a successful infection. It remains a possibility that the time of infection influenced the blood feeding success rate as some species of mosquitoes are known to blood feed (i.e., host-seek) at particular times of day [50]. Moreover, despite the differences in feeding rates among the species and between the methods, side-by-side experiments showed no significant differences between the rates of infection, dissemination, and transmission. Sugar infections can be standardized across large cohorts removing the need for host cues and enabling reproducible and statistically robust experiments even in resource-limited settings. The short exposure periods required to achieve infection further demonstrate its practicality for vector competence studies.

WNV is a widely studied arbovirus, and several studies have demonstrated the vector competence of *Cx. quinquefasciatus* for the virus [42,51–53]. Infection and dissemination rates of laboratory colonies infected using artificial blood meals ranged from 52% (IR) and 22% (DR) at 28°C (13 dpi) to 100% (IR) and 73% (DR) at 28°C (12 dpi) in the literature [53,54]. Richards et al. [52] compared susceptibility between two *Cx. quinquefasciatus* colonies, observing infection rates of 81% and 69% at 28°C (13 dpi), with the dissemination rates being 64% and 79%, respectively. Studies that used wild-caught strains (i.e., field populations) of *Cx. quinquefasciatus* reported WNV infection rates ranging between 38.7-100% at 28°C (12–14 dpi) and dissemination rates ranging between 22.6-60% [42,55,56]. We observed a high infection rate (97.2%) and dissemination rate (97.1%) with *Cx. quinquefasciatus* after only 10 dpi at 27°C, which aligns with other studies, although the DR observed in our study was higher than those reported previously. *Aedes albopictus* is also a known vector of WNV and has been found to be naturally infected with WNV in North America [57]. Sardelis et al. (2002) [58] investigated the vector competence of four strains of *Ae. albopictus* found that at 27°C (14 dpi) the infection and dissemination rates ranged between 56–96% and 49–93%, respectively. Others report WNV infection and dissemination rates ranging between 80–90% and 50–85%, respectively [59,60]; however, there is significant variability in the methodologies of these studies. Following an IVSM, we observed infection rates between 88.2-100% and dissemination rates between 90–100% in *Ae. albopictus*. Again, in line with the other mosquito species, our results were in keeping with the high-end range of previous studies using two methods of infection.

While WNV was detected in all trials for both *Cx. quinquefasciatus* and *Ae. albopictus* mosquitoes, it is important to note that forced salivation (FS) using immersion oil has limitations that can reduce the amount of virus recovered from capillary tubes [61]. While pooling samples can increase the quantity of virus [62], it was not applicable to single-mosquito screening; thus, it remained difficult to determine whether negative readings resulted from uninfected salivary glands within the female or because a sample was not fully retrieved from the capillary tube [61]. Transmission potential has also been shown to differ between *in vitro* methods, such as capillary tube collections, and the use of animals [63]. For example, *Cx. pipiens* transmitted more WNV to animals compared to capillary tubes, whereas *Ae. albopictus* transmitted more Venezuelan equine encephalitis virus via capillary tubes than in animal models [63–65]. Nonetheless, we observed transmission rates of 14.3%-21.4% and 45.5%-50% in *Cx. quinquefasciatus* after 7 and 10 dpi, respectively; and 7.4%-28.6% and 21.4%-22.2% in *Ae. albopictus* after 7 and 10 dpi, respectively. Previous findings from Anderson et al. (2010) showed that after 13 and 14 dpi, 13% (3/13) and 29% (29/100) of *Cx. quinquefasciatus* saliva samples were positive for WNV (WN-FL03p2–3) following capillary tube collection with immersion oil [66]. Conversely, Vanlandingham et al. (2004) found that after 14 dpi, 87% (46/53) of *Cx. quinquefasciatus* saliva samples were positive for WNV (strain homologous to NY99), but using capillary tubes filled with Nujol mineral oil [67]. Both *Ae. albopictus* and *Cx. quinquefasciatus* became efficiently infected with WNV following an infectious sugar meal, confirming that virus ingested through sucrose solutions can escape from the digestive tract and reach secondary tissues. The progression of infection across BD, LW, and SL tissues mirrored the canonical sequence of midgut invasion, dissemination through the hemocoel, and eventual salivary gland infection, as seen in other vector competence studies [68], supporting the evidence shown here that the IVSM technique may serve a physiologically authentic route of infection.

Regarding MAYV, our study demonstrated that *Ae. albopictus* can be infected via an infectious sugar meal, with an infection rate ranging from 86.2% to 96.8% after 7 dpi and reaching 100% after 10 dpi. Dissemination was very high in three of the four experiments, ranging from 81.8% to 94.3% after 10 dpi. A lower dissemination rate was observed at 7 dpi after the 1 hr feeding (36%) compared to the 24 hr feeding (83.3%), which may be attributed to an outlier mosquito batch in one of the three 1 hr trials, lowering the overall DR at this timepoint. Pereira et al. (2020) [23] reported infection rates of 61.6% in *Ae. albopictus* collected in Brazil, which is lower than three out of the four MAYV infection trials we observed. Brustolin et al. (2024) [69] assessed the vector competence of *Ae. albopictus* from Italy, achieving a 90.9% IR and 63.3% DR at 7 dpi, and 91.7% IR and 90.9% DR at 10 dpi, aligning very closely with our results. *Culex quinquefasciatus*, conversely, was unable to maintain viral replication or transmit the virus, remaining refractory to MAYV. Infection rate ranged from 10.7% to 17.2% but decreased in both experiments between 7 and 10 dpi, respectively, whereas dissemination rate ranged from 33.3% (2/3) to 80% (4/5) between 7 and 10 dpi, respectively. But it should be noted that the DR percentages were dependent on the number of positive BD tissues, thereby, greatly inflating the rate of dissemination for *Cx. quinquefasciatus*. Moreover, after a 24 hr feeding and 10 dpi, no positive samples were observed for the BD and LW, further indicating refraction over time. Pereira et al. (2020) and Krokovsky et al. (2023) [70] also showed very low competence of *Cx. quinquefasciatus* to MAYV, with reported infection rates being 2.5% and 13–15%, respectively. In our study, MAYV transmission was low in *Ae. albopictus*, ranging between 4-24.2%, but MAYV was only detected in one *Cx. quinquefasciatus* sample out of the 114 females processed. At 14 dpi, Fernández et al. (2023) observed MAYV transmission rates at 16.7% (MAYV2668) and 25% (MAYV0179) using capillary tubes filled with FBS in *Ae. albopictus* originating from Tapachula, Mexico [71]. Wiggins et al. (2018) used a combination of immersion oil-filled capillary tubes and cationic papers to assess MAYV (TRVL4675) transmission in *Ae. albopictus* and observed overall low transmission rates throughout the infection period, which peaked at 55% at 9 dpi and then decreased to 28% at 12 dpi [72]. Our findings of 24.2% transmission rate at 10 dpi (1 hr) align with both Fernández et al. (2023) and Wiggins et al. (2018) at 12 dpi.

The results associated with ZIKV infections in *Ae. albopictus* do not corroborate the studies conducted by Gloria-Soria et al. (2020) [73] and Azar et al. (2017) [74]. In these studies, the infection and dissemination rates found for ZIKV ranged from 63-98% (IR) and 15–98% (DR) to 100% (IR) and ~45–90% (DR), while the results of our study using IVSM ranged from 5.9-13.8% (IR) and 25–50%% (DR), respectively. However, we highlight that, with a very small number of samples positive for BD tested for DR, this value may not be representative. While Gloria-Soria et al. (2020) reported a few mosquitoes with detectable virus in their saliva and Azar et al. (2017) showed more efficient viral shedding into the saliva, in our study, between the 122 *Ae. albopictus* tested for ZIKV (PRVABC59) and 54 tested for ZIKV (R116265), zero samples were positive from saliva. Despite these two studies using different *Ae. albopictus* strains and methods of infection (i.e., artificial blood meal and infectious host animal), they use the same ZIKV (PRVABC59) strain used throughout our study, but with different outcomes. In addition, the titer of ZIKV (PRVACB59) ranged from 2.0 to 7.9 × 10^7^ PFU/mL [73], over a ten-fold greater titer than that achieved in this study (1.9 × 10^6^ PFU/mL) which, in conjunction with the days examined, may have contributed to the low IR observed. Another study that examined vector potential of *Ae. albopictus* for ZIKV (SL1602) observed that a titer of 1.9 to 6.5 × 10^5^ FFU/mL was sufficient to infect 50% of the ZIKV exposed mosquitoes (ID50) [75]. In the same study, when exposed to 3 × 10^6^ FFU/mL, between 60% and 95% of BD were infected by 10 dpi which was maintained up to 20 dpi [75]. In comparison, after both 1 hr and 24 hr feedings, our IR never surpassed 15% at either 7 dpi (5.9-12.9%) or 10 dpi (7.1-13.8%) despite feeding with a titer of 1.9 × 10^6^ PFU/mL, indicating a strain-host interaction which may cause a delay in infection or rather indicating ZIKV refraction. To determine if the *Ae. albopictus* were not competent for the PRVABC59 strain, and explore the strain-host interaction, we performed IVSMs with the R116265 strain, resulting in only one positive BD and LW sample (at 10 dpi) – again, suggesting a potential refraction or substantially delayed infection to ZIKV. Despite being known as efficient ZIKV vectors in nature, *Ae. albopictus* have previously demonstrated variable, and at times, poor vector competence for the virus under laboratory conditions [74,76,77]. Regarding the results found in *Cx. quinquefasciatus*, some research groups from China, Brazil, and the USA [27–29] were able to artificially infect the species with ZIKV. The literature is discrepant regarding the vector competence of *Cx. quinquefasciatus* and ZIKV, with much of the data showing a lack of ability to become infected with and transmit the virus [78–81]. In this study, both mosquito species showed little to no ZIKV infectivity, with *Ae. albopictus* showing refraction to a second strain from a different geographical origin. Moreover, side-by-side blood infections also resulted in similar low outcomes of ZIKV infections, which raises the question whether the two mosquito strains from New Jersey, USA (*Ae. albopictus*), and Johannesburg, South Africa (*Cx. quinquefasciatus*) are not competent for ZIKV strains originating in the Americas. Factors such as the use of fresh versus frozen viral stocks, titer, ambient temperature effects on incubation periods, and the duration of the infection period can also influence, or even impair, mosquito infectivity with ZIKV [82–85], all of which could explain the lack of infection we observed in both IVSM and blood feeding experiments.

Although the physiological pathway by which the virus establishes infection following sugar ingestion is not yet fully defined, our results provide initial evidence of efficient viral uptake and systemic dissemination. In mosquitoes, ingested sugar is first stored in the crop – a diverticulum connected to the foregut – before gradually entering the midgut for digestion [86,87]. The virus may potentially infect epithelial cells in either tissue, before disseminating throughout the mosquito – a route future studies should investigate to provide further mechanistic insights into infections with sugar meals. From an ecological standpoint, sugar feeding is a near-universal behavior among mosquitoes and occurs far more frequently than blood feeding [86]. It plays a crucial role in energy metabolism, survival, and mating behavior [38]. Our findings extend this concept experimentally, showing that sugar meals can facilitate arbovirus acquisition and replication earlier after emergence without the need for sexual maturity or mating, thus enabling prolonged experiments. The IVSM technique has also been effectively implemented with wild-caught mosquitoes and has demonstrated successful ingestion and infection in populations that may be reluctant to blood feed in a laboratory environment. These features make IVSM a powerful model for exploring virus–vector interactions under conditions approximating natural feeding behavior.

While IVSM shows strong potential, some limitations warrant further study. First, the physiological route of infection from the crop to the midgut epithelium remains to be elucidated. It is unclear whether the virus replicates within the crop epithelium before entering the hemocoel or translocates to the midgut prior to dissemination, the latter of which would mimic infection via blood meal. Histological and *in situ* hybridization studies could clarify these pathways and quantify viral tropism across tissues [88]. Second, although IVSM reproduces infection patterns such as those observed with blood feeding, viral titers in sugar solutions may degrade over time, depending on the virus, potentially influencing infection efficiency during prolonged exposure. We assessed the viral titer of a subset of sugar meals after mosquito feeding and observed decline in titer compared to the original sugar meal (S2 Table). However, they remained sufficiently infectious to yield suitably high infection rates (S3 Table), with perhaps an exception in the case of ZIKV as discussed earlier. Future work should verify infectivity for different viruses, mosquito species, and/or feeding times to optimize IVSM conditions. Ultimately, forced salivation does not accurately reflect arboviral transmission [76]. This inaccuracy can be attributed to several factors, including low viral titers in saliva, variability among populations, potential re-ingestion of saliva during FS, and the fact that mosquitoes selectively secrete enzymes that differ depending on whether they are blood- or sugar-feeding [76,89–91]. Transmission success from IVSM-infected mosquitoes should be validated through live-virus transmission assays or vector-to-host challenge models to confirm epidemiological relevance. It should be noted that previous research by Almire et al., (2021) showed that sugar feeding changes the antiviral states of the digestive tract of *Ae. aegypti*, thereby altering susceptibility to Semliki Forest (SFV) virus and ZIKV [92]. While *Ae. aegypti* were primed with sugar 16 hrs before a subsequent infectious blood meal, the effects on SFV and ZIKV differed, in that SFV showed lower viral titers, whereas ZIKV had a reduced proportion of infected mosquitoes. In IVSM, however, sugar and virus are ingested together, and sugar digestion (i.e., green dye in the midgut and reduced crop size, or green waste droplets) was apparent as early as 2 hrs post-IVSM. Therefore, the effect of sugar-enhanced immunity may not be as clear as seen by Almire et al., (2021) [92] and would require further investigation in the context of IVSM. *Aedes aegypti* are highly anthropophilic and have been shown capable of surviving and reproducing while feeding on human blood alone [93,94], and while Braks et al., (2006) also showed *Ae. albopictus* can be maintained on a diet of only human blood, sugar-enhanced immunity has only been shown in *Ae. aegypti.* Therefore, further research to determine whether the sugar-enhanced immunity phenomenon is unique to *Ae. aegypti*, or whether it is conserved across other *Aedes* spp., or culicid genera, could provide more insight into the mechanism of the IVSM technique.

It should be noted that comparing previously published studies on vector competence of mosquito species for specific arboviruses with the results obtained using the IVSM technique is challenging due to substantial heterogeneity in experimental designs, laboratory conditions, and analytical methods across the literature. Factors such as virus strain (including lineage, storage, and inoculum dose), mosquito species and population origins, and mosquito handling practices (age at infection, rearing history, and environmental conditions) can all influence infection, dissemination, and transmission outcomes [68,84]. Furthermore, differences in experimental methodology, such as the mosquito route of infection (oral blood meal versus intrathoracic inoculation), incubation temperature and infection duration, saliva collection media and timing, and detection technology (plaque assays versus RT-qPCR), combined with variable criteria for presenting results (i.e., positivity versus rate), further complicate cross-study comparisons [68,84]. Despite no significant differences between side-by-side IVSM and blood feeding experiments, these biological and methodological sources of variability must be considered when evaluating IVSM-based data across studies, highlighting the need for standardized experimental approaches.

Our results show that this method is efficient for studying vector competence in mosquitoes. However, further optimization is required, and comprehensive comparative studies reproducing the conditions of previous vector competence studies (e.g., mosquito strain, virus strain, temperature, infection period, etc.)—with the sole substitution of infectious sugar meals for blood—are essential to accurately assess the capabilities and the limitations of the IVSM method. The infection via sugar meal (IVSM) technique represents a valuable methodological advancement in arboviral research. Our data confirm that sugar feeding can serve as a reliable, efficient, and reproducible route for arbovirus infection, particularly for viruses like WNV and MAYV. As arboviral diversity and vector geographic ranges continue to expand globally, this method provides an adaptable framework for investigating virus-vector interactions, co-infections, and potential intervention strategies under risk-reduced and reproducible laboratory conditions. IVSM could offer a powerful new framework to accelerate discovery and enhance preparedness for emerging vector-borne diseases.

## Supporting information

S1 Table Direct-to-PCR versus extraction Cq values run in duplicate for MAYV and ZIKV. A side-by-side comparison of RNA extraction compared to direct sample addition and heat inactivation (95°C for 15 mins) of serially diluted virus samples. ND (not determined) indicates no signal amplification during the run for the specified sample.
(DOCX)

S2 Table ZIKV IVSM titers pre- and post-feeding as determined by plaque assay in Vero cells. Zika virus sugar meal titers at time zero, and following a 1 hr and 24 hr IVSM, confirmed with cytopathic effect (CPE) in Vero cells and tittered via plaque assay.
(DOCX)

S3 Table Determination of infectivity of sugar meals pre- and post-feeding in Vero cell culture. Cytopathic effect (CPE) in Vero cells was observed through a media color change from red to yellow alongside microscopic evaluation and confirmation of cell rounding and detachment.
(DOCX)

S1 Data WNV RNA copy number/mL in *Ae. albopictus* and *Cx. quinquefasciatus* after a 1 hr and 24 hr IVSM collected at 7 and 10 dpi. RNA copy numbers in mosquito bodies (BD), legs and wings (LW), and saliva (SL), were used to determine the rates of West Nile virus infection, dissemination, and transmission, respectively. ND indicates no signal amplification during the run for the specified sample.
(DOCX)

S2 Data MAYV RNA copy number/mL in *Ae. albopictus* and *Cx. quinquefasciatus* after a 1 hr and 24 hr IVSM collected at 7 and 10 dpi. RNA copy numbers in mosquito bodies (BD), legs and wings (LW), and saliva (SL), were used to determine the rates of Mayaro virus infection, dissemination, and transmission, respectively. ND indicates no signal amplification during the run for the specified sample.
(DOCX)

S3 Data ZIKV RNA copy number/mL in *Ae. albopictus* and *Cx. quinquefasciatus* after a 1 hr and 24 hr IVSM collected at 7 and 10 dpi. RNA copy numbers in mosquito bodies (BD), legs and wings (LW), and saliva (SL), were used to determine the rates of Zika virus infection, dissemination, and transmission, respectively. ND indicates no signal amplification during the run for the specified sample.
(DOCX)

S4 Data WNV RNA copy number/mL in *Ae. albopictus* and *Cx. quinquefasciatus* after side-by-side IVSM and blood feeding infections. RNA copy numbers in mosquito bodies (BD), legs and wings (LW), and saliva (SL), were used to determine the rates of West Nile virus infection, dissemination, and transmission, respectively. Females were infected at 2 and 7 days-post-eclosion (dpe) for 1 hr and collected at 10 dpi. ND indicates no signal amplification during the run for the specified sample.
(DOCX)
