# Supplementary material for: Development of an infection via sugar meal (IVSM) technique to infect mosquitoes (Diptera: Culicidae) with arthropod-borne viruses
Source: PLoS Negl Trop Dis. 2026 Nov 2;20(11):e0014715. doi: 10.1371/journal.pntd.0014715 (PMC13750647; doi:10.1371/journal.pntd.0014715)
Supplement: Supplementary file 1 [file pntd.0014715.s001.docx]

**S1 Table. Direct-to-PCR versus extraction Cq values run in duplicate for MAYV and ZIKV.**

| **Method** | **Titer** | **MAYV** | |  | **ZIKV (PRVABC5)** | |  | **ZIKV (R116265)** | |
| --- | --- | --- | --- | --- | --- | --- | --- | --- | --- |
| Extraction | 10^6^ | ND | 15.69 |  | 19.15 | 18.58 |  | 19.33 | 20.68 |
|  | 10^5^ | 19.06 | 20.16 |  | 23.74 | 23.41 |  | 26.05 | 25.04 |
|  | 10^4^ | 22.81 | 24.20 |  | 28.59 | 25.61 |  | 26.89 | 26.42 |
|  | 10^3^ | 25.91 | 28.38 |  | 30.28 | 28.04 |  | 29.89 | 29.18 |
|  | 10^2^ | 31.98 | 31.42 |  | 32.11 | 31.85 |  | 31.65 | 32.56 |
|  | 10^1^ | 36.10 | 35.01 |  | 36.00 | 34.38 |  | 35.55 | 36.13 |
| Direct-to-PCR | 10^6^ | 17.37 | 18.00 |  | 19.26 | 19.75 |  | 18.95 | 19.47 |
|  | 10^5^ | 19.17 | 19.64 |  | 23.33 | 24.00 |  | 23.03 | 22.74 |
|  | 10^4^ | 23.36 | 23.39 |  | 26.40 | 26.22 |  | 25.57 | 25.71 |
|  | 10^3^ | 28.00 | 27.83 |  | 29.63 | 30.23 |  | 29.82 | 30.30 |
|  | 10^2^ | 31.72 | 32.05 |  | 33.13 | 32.50 |  | 33.36 | 33.04 |
|  | 10^1^ | 33.89 | ND |  | 35.83 | 35.64 |  | 36.97 | 37.07 |
