# Supplementary material for: Development of an infection via sugar meal (IVSM) technique to infect mosquitoes (Diptera: Culicidae) with arthropod-borne viruses
Source: PLoS Negl Trop Dis. 2026 Nov 2;20(11):e0014715. doi: 10.1371/journal.pntd.0014715 (PMC13750647; doi:10.1371/journal.pntd.0014715)
Supplement: Supplementary file 2 [file pntd.0014715.s002.docx]

**S2 Table. ZIKV IVSM titers pre- and post-feeding as determined by plaque assay in Vero cells.**

| **Virus** | **Mosquito** | **Feeding Time** | **CPE Observed** | **Titer (PFU/mL)** |
| --- | --- | --- | --- | --- |
| ZIKV | *Culex* | 0-hr | Yes | 1.93 x 10^6^ |
|  |  | 1-hr | Yes | 6.25 x 10^5^ |
|  |  | 24-hr | Yes | 3.10 x 10^5^ |
| ZIKV | *Culex* | 0-hr | Yes | 1.91 x 10^6^ |
|  |  | 1-hr | Yes | 6.00 x 10^5^ |
|  |  | 24-hr | Yes | 2.50 x 10^5^ |
