# Supplementary material for: Development of an infection via sugar meal (IVSM) technique to infect mosquitoes (Diptera: Culicidae) with arthropod-borne viruses
Source: PLoS Negl Trop Dis. 2026 Nov 2;20(11):e0014715. doi: 10.1371/journal.pntd.0014715 (PMC13750647; doi:10.1371/journal.pntd.0014715)
Supplement: Supplementary file 3 [file pntd.0014715.s003.docx]

**S3 Table. Determination of infectivity of sugar meals pre- and post-feeding in Vero cell culture.**

| **Virus** | **Mosquito** | **Feeding Time (hr)** | **CPE Observed** | **PCR confirmation** |
| --- | --- | --- | --- | --- |
| WNV | *Aedes, Culex* | 0 | Yes | Yes |
|  |  | 1 | Yes | Yes |
|  |  | 24 | Yes | Yes |
| MAYV | *Aedes, Culex* | 0 | Yes | Yes |
|  |  | 1 | Yes | Yes |
|  |  | 24 | Yes | Yes |
| ZIKV | *Aedes, Culex* | 0 | Yes | Yes |
|  |  | 1 | Yes | Yes |
|  |  | 24 | Yes | Yes |

*CPE observed: media color change from red to yellow and microscopic evaluation of cell rounding and detachment.
