# Supplementary material for: Development of an infection via sugar meal (IVSM) technique to infect mosquitoes (Diptera: Culicidae) with arthropod-borne viruses
Source: PLoS Negl Trop Dis. 2026 Nov 2;20(11):e0014715. doi: 10.1371/journal.pntd.0014715 (PMC13750647; doi:10.1371/journal.pntd.0014715)
Supplement: Supplementary file 4 [file pntd.0014715.s004.docx]

| **Table A.** WNV RNA copy number/mL in *Ae. albopictus* after a 1 hr IVSM at 7 and 10 dpi. | | | | | | |
| --- | --- | --- | --- | --- | --- | --- |
| **Sample** | **7 dpi** | | | **10 dpi** | | |
|  | BD | LW | SL | BD | LW | SL |
| 1 | 4.15E+10 | 1.76E+08 | ND | 5.91E+05 | 1.06E+05 | ND |
| 2 | 6.72E+06 | 1.30E+07 | ND | 1.48E+09 | 3.04E+07 | ND |
| 3 | 1.58E+10 | 2.88E+08 | ND | 3.68E+10 | 9.34E+05 | ND |
| 4 | 4.50E+05 | 2.23E+05 | ND | 2.39E+05 | 1.52E+05 | ND |
| 5 | 4.69E+04 | ND | ND | 2.36E+10 | 6.88E+06 | ND |
| 6 | 7.78E+09 | 9.33E+06 | ND | 2.72E+10 | 8.90E+05 | ND |
| 7 | 2.32E+09 | 1.05E+05 | ND | 9.43E+09 | 1.38E+06 | ND |
| 8 | 1.44E+05 | 1.07E+05 | ND | 1.65E+10 | 1.05E+06 | ND |
| 9 | 3.23E+08 | 8.58E+04 | ND | 1.27E+06 | 1.85E+05 | ND |
| 10 | 2.32E+11 | 3.88E+09 | 8.89E+04 | 2.68E+10 | 1.72E+05 | ND |
| 11 | 2.05E+07 | 1.41E+05 | ND | 7.25E+10 | 3.58E+08 | 6.83E+05 |
| 12 | ND | ND | ND | 6.13E+11 | 9.55E+09 | 6.78E+07 |
| 13 | 1.84E+10 | 1.32E+08 | ND | 8.83E+06 | 9.95E+05 | ND |
| 14 | 8.46E+04 | 1.18E+05 | ND | 5.33E+05 | 1.09E+06 | ND |
| 15 | 1.60E+10 | 9.92E+04 | ND | 1.37E+05 | 2.52E+05 | ND |
| 16 | 3.13E+05 | 1.24E+05 | ND | ND | ND | ND |
| 17 | 1.50E+10 | 6.37E+07 | ND | 1.83E+10 | 1.18E+07 | 1.68E+05 |
| 18 | 2.18E+05 | ND | ND | 2.65E+05 | 6.07E+07 | ND |
| 19 | 2.97E+05 | 4.31E+04 | ND | 2.35E+05 | 3.01E+06 | ND |
| 20 | 2.11E+07 | 6.64E+04 | ND | 4.48E+11 | 8.32E+09 | 4.71E+06 |
| 21 | 2.79E+10 | 8.58E+08 | ND | 1.43E+11 | 2.72E+09 | ND |
| 22 | 1.06E+06 | 2.28E+08 | ND | 3.32E+11 | 5.88E+09 | ND |
| 23 | 7.82E+04 | 1.61E+07 | ND | 1.65E+10 | 3.22E+08 | ND |
| 24 | 4.84E+09 | 3.64E+05 | ND | 2.31E+10 | 5.46E+09 | 1.68E+07 |
| 25 | 3.20E+05 | ND | ND | 6.09E+05 | 5.98E+05 | ND |
| 26 | ND | ND | ND | 3.01E+05 | 1.21E+06 | ND |
| 27 | 1.05E+05 | 2.25E+05 | ND | 4.35E+05 | 2.85E+06 | ND |
| 28 | ND | ND | ND | 2.08E+12 | 5.36E+09 | 3.71E+07 |
| 29 | ND | ND | ND | - | - | - |
| 30 | 1.07E+10 | 1.41E+08 | ND | - | - | - |
| 31 | 2.94E+10 | 9.69E+08 | 8.41E+05 | - | - | - |
| 32 | 1.28E+10 | 1.33E+08 | ND | - | - | - |
| 33 | 1.31E+06 | 3.23E+05 | ND | - | - | - |
| 34 | 9.25E+05 | 1.07E+06 | ND | - | - | - |

| **Table B.** WNV RNA copy number/mL in *Ae. albopictus* after a 24 hr IVSM at 7 and 10 dpi. | | | | | | |
| --- | --- | --- | --- | --- | --- | --- |
| **Sample** | **7 dpi** | | | **10 dpi** | | |
|  | BD | LW | SL | BD | LW | SL |
| 1 | 1.84E+06 | 8.09E+06 | ND | 1.71E+11 | 7.41E+08 | ND |
| 2 | 5.88E+05 | 6.85E+05 | ND | 5.04E+05 | 3.40E+05 | ND |
| 3 | 1.12E+06 | 2.06E+07 | ND | 6.33E+11 | 2.31E+09 | 3.46E+07 |
| 4 | 9.82E+05 | 3.47E+06 | ND | 8.18E+05 | 1.99E+06 | ND |
| 5 | 1.16E+12 | 3.07E+10 | 5.69E+04 | 3.69E+11 | 1.50E+09 | ND |
| 6 | 1.06E+12 | 2.64E+10 | 2.57E+04 | 7.61E+11 | 3.85E+09 | ND |
| 7 | 1.07E+07 | 2.06E+07 | ND | 1.16E+06 | 8.71E+05 | ND |
| 8 | 3.41E+11 | 1.36E+10 | ND | 2.14E+05 | 1.23E+05 | ND |
| 9 | 1.02E+12 | 2.40E+10 | ND | 1.15E+06 | 1.87E+05 | ND |
| 10 | 6.33E+06 | 5.01E+09 | 9.63E+04 | 3.58E+05 | 2.33E+05 | ND |
| 11 | 1.45E+12 | 1.08E+10 | 5.81E+06 | 2.95E+11 | 1.45E+09 | ND |
| 12 | 1.39E+06 | 1.47E+07 | 5.75E+04 | 4.11E+11 | 2.81E+09 | ND |
| 13 | 8.55E+10 | 4.07E+08 | ND | 6.17E+11 | 9.94E+09 | ND |
| 14 | 1.24E+12 | 7.27E+09 | 5.98E+05 | 9.42E+11 | 5.34E+09 | 8.84E+05 |
| 15 | 2.29E+11 | 3.52E+06 | ND | 4.71E+05 | 8.18E+05 | ND |
| 16 | 3.46E+06 | 3.26E+06 | ND | 8.57E+11 | 2.33E+09 | ND |
| 17 | 1.30E+07 | 1.52E+06 | ND | 1.01E+06 | 9.70E+05 | ND |
| 18 | 1.16E+12 | 2.20E+10 | ND | 1.48E+06 | 1.49E+05 | ND |
| 19 | 1.17E+06 | 3.74E+06 | ND | 3.03E+05 | 3.28E+05 | 4.34E+04 |
| 20 | 7.99E+10 | 2.10E+07 | ND | 3.40E+05 | 1.32E+05 | ND |
| 21 | 1.13E+06 | 4.63E+06 | ND | 4.60E+06 | 2.33E+06 | ND |
| 22 | 5.59E+11 | 1.89E+09 | ND | 1.80E+05 | 1.64E+05 | ND |
| 23 | 2.92E+11 | 7.59E+09 | 3.82E+06 | 2.44E+05 | 1.03E+05 | ND |
| 24 | 6.06E+05 | 9.25E+07 | ND | 2.25E+05 | 1.09E+06 | ND |
| 25 | 3.49E+12 | 1.58E+10 | 1.76E+05 | 1.10E+12 | 4.78E+09 | 8.56E+04 |
| 26 | 1.70E+06 | 2.05E+07 | ND | 1.58E+08 | 1.50E+05 | ND |
| 27 | 3.13E+06 | 3.86E+06 | ND | 7.45E+11 | 3.77E+09 | 8.49E+05 |
| 28 | 2.39E+10 | 8.88E+06 | ND | 7.69E+11 | 3.57E+09 | 1.48E+07 |

| **Table C.** WNV RNA copy number/mL in *Cx. quinquefasciatus* after a 1 hr IVSM at 7 and 10 dpi. | | | | | | |
| --- | --- | --- | --- | --- | --- | --- |
| **Sample** | **7 dpi** | | | **10 dpi** | | |
|  | BD | LW | SL | BD | LW | SL |
| 1 | 4.89E+05 | 5.57E+05 | ND | 1.21E+11 | 5.73E+08 | 1.02E+04 |
| 2 | 4.41E+05 | ND | ND | 9.99E+06 | 4.09E+05 | ND |
| 3 | 1.13E+05 | 1.36E+05 | ND | 1.18E+11 | 4.67E+08 | 8.67E+04 |
| 4 | 2.15E+11 | 3.36E+09 | 3.51E+06 | 9.83E+09 | 2.68E+05 | ND |
| 5 | 2.76E+05 | ND | ND | 8.94E+10 | 7.16E+06 | 1.44E+05 |
| 6 | 1.06E+06 | 2.48E+05 | ND | 1.03E+10 | 1.18E+07 | 8.66E+04 |
| 7 | ND | ND | ND | 2.21E+10 | 2.15E+08 | ND |
| 8 | 8.24E+05 | 7.82E+04 | ND | 1.06E+11 | 1.62E+05 | ND |
| 9 | 1.75E+07 | 5.01E+05 | 1.32E+06 | 6.41E+10 | 3.99E+08 | ND |
| 10 | ND | ND | ND | 1.55E+05 | 3.30E+06 | 2.29E+04 |
| 11 | 8.82E+08 | 1.40E+05 | ND | 1.88E+11 | 1.08E+09 | 2.50E+06 |
| 12 | 3.91E+05 | 4.41E+05 | ND | 1.80E+05 | 5.22E+05 | ND |
| 13 | 8.39E+05 | 6.99E+05 | ND | 3.63E+06 | 1.11E+06 | ND |
| 14 | ND | ND | ND | 6.31E+09 | 1.78E+07 | ND |
| 15 | 9.32E+05 | 1.88E+05 | ND | 5.14E+10 | 3.92E+08 | 1.65E+05 |
| 16 | 5.01E+09 | ND | ND | 3.98E+10 | 6.07E+07 | 8.00E+03 |
| 17 | 3.54E+09 | 2.57E+07 | 3.65E+06 | 3.03E+05 | 1.52E+06 | ND |
| 18 | ND | ND | ND | 3.62E+10 | 6.32E+08 | 4.47E+04 |
| 19 | ND | ND | ND | 3.04E+10 | 2.82E+08 | 2.59E+05 |
| 20 | 2.22E+05 | 7.68E+04 | ND | 6.79E+10 | 1.99E+09 | 9.62E+05 |
| 21 | 3.55E+05 | 1.76E+05 | ND | 2.43E+06 | 9.71E+05 | ND |
| 22 | 5.11E+05 | 8.03E+04 | ND | 6.44E+04 | 4.87E+06 | ND |
| 23 | ND | ND | ND | 9.36E+10 | 6.12E+08 | ND |
| 24 | 4.76E+09 | 2.79E+05 | ND | 7.24E+10 | ND | ND |
| 25 | 3.82E+05 | 5.49E+04 | ND | 4.83E+11 | 2.44E+09 | 1.12E+05 |
| 26 | 4.23E+05 | 1.11E+05 | ND | 3.44E+11 | 5.54E+08 | 2.50E+07 |
| 27 | ND | ND | ND | 2.35E+11 | 3.88E+08 | 2.20E+05 |
| 28 | 2.17E+06 | 1.51E+05 | ND | 3.47E+05 | 7.29E+04 | ND |
| 29 | 4.64E+04 | ND | ND | 8.82E+10 | 1.23E+09 | ND |
| 30 | 1.26E+06 | 6.92E+05 | ND | 4.04E+11 | 2.47E+09 | 1.30E+05 |
| 31 | 4.92E+05 | 6.50E+05 | ND | 2.12E+11 | 1.76E+09 | 1.28E+05 |
| 32 | 4.00E+08 | ND | ND | 1.06E+05 | 3.96E+05 | ND |
| 33 | 1.19E+06 | 4.31E+05 | ND | ND | ND | ND |
| 34 | - | - | - | 6.04E+07 | 4.79E+05 | ND |
| 35 | - | - | - | 4.02E+11 | 4.81E+09 | 2.15E+05 |
| 36 | - | - | - | 2.04E+11 | 9.53E+08 | ND |

| **Table D.** WNV RNA copy number/mL in *Cx. quinquefasciatus* after a 24 hr IVSM at 7 and 10 dpi. | | | | | | |
| --- | --- | --- | --- | --- | --- | --- |
| **Sample** | **7 dpi** | | | **10 dpi** | | |
|  | BD | LW | SL | BD | LW | SL |
| 1 | 2.01E+07 | 4.45E+07 | ND | 6.57E+08 | 4.05E+09 | 2.53E+06 |
| 2 | 3.76E+11 | 2.37E+08 | 1.27E+05 | 5.90E+05 | 3.02E+04 | ND |
| 3 | 5.34E+10 | 1.34E+08 | 1.45E+05 | 7.83E+06 | 1.07E+05 | ND |
| 4 | 7.50E+04 | 2.21E+04 | ND | 2.65E+10 | 5.25E+08 | 3.37E+04 |
| 5 | ND | ND | ND | 5.68E+04 | 3.71E+04 | ND |
| 6 | 1.27E+10 | 4.68E+04 | ND | 8.32E+10 | 3.48E+08 | 1.99E+06 |
| 7 | 5.13E+04 | 2.21E+08 | ND | 5.18E+05 | ND | ND |
| 8 | ND | ND | ND | 1.11E+06 | 4.78E+04 | ND |
| 9 | 4.17E+04 | 4.63E+04 | ND | 1.39E+11 | 2.34E+09 | 9.19E+04 |
| 10 | ND | ND | ND | 5.17E+06 | 7.38E+04 | ND |
| 11 | 5.37E+08 | 1.02E+05 | ND | 9.68E+04 | 3.44E+06 | 1.52E+05 |
| 12 | ND | ND | ND | 2.12E+07 | 3.30E+06 | 4.29E+05 |
| 13 | 1.41E+11 | 1.34E+07 | 4.03E+06 | 4.41E+05 | 7.38E+04 | ND |
| 14 | ND | ND | ND | 1.28E+05 | 1.38E+05 | ND |
| 15 | ND | ND | ND | 7.76E+10 | 1.80E+09 | 1.58E+06 |
| 16 | 6.26E+04 | 1.04E+05 | ND | 3.27E+05 | 9.05E+04 | ND |
| 17 | 1.38E+05 | 4.07E+05 | ND | 8.17E+05 | 2.81E+05 | ND |
| 18 | 1.25E+10 | 1.24E+05 | ND | 6.64E+10 | 6.95E+08 | 3.26E+04 |
| 19 | 1.30E+11 | 8.64E+07 | ND | 8.51E+10 | 1.29E+09 | 2.35E+04 |
| 20 | 4.86E+04 | 9.25E+04 | ND | 1.22E+10 | 2.03E+08 | ND |
| 21 | ND | ND | ND | 4.07E+05 | 1.10E+06 | ND |
| 22 | 5.58E+04 | 1.65E+05 | ND | 6.30E+10 | 1.19E+09 | 2.22E+05 |
| 23 | 2.26E+05 | 3.57E+04 | ND | 1.72E+11 | 3.53E+05 | ND |
| 24 | 1.61E+05 | 5.20E+04 | ND | 5.62E+05 | 4.15E+05 | ND |
| 25 | 2.44E+11 | 3.93E+08 | 8.13E+05 | 6.52E+10 | 2.03E+09 | 1.79E+06 |
| 26 | 4.12E+05 | 7.40E+04 | ND | 1.32E+10 | 2.98E+05 | ND |
| 27 | 1.00E+05 | 3.05E+05 | ND | 7.40E+10 | 2.37E+09 | 3.25E+06 |
| 28 | ND | ND | ND | 1.49E+10 | 5.64E+04 | ND |
| 29 | 9.06E+04 | 3.74E+05 | ND | 1.26E+05 | 4.76E+04 | ND |
| 30 | 7.84E+09 | 1.01E+09 | 3.63E+06 | 1.44E+05 | 5.67E+04 | ND |
| 31 | 4.46E+07 | 4.14E+04 | ND | 1.26E+05 | 2.73E+05 | ND |
| 32 | 5.66E+05 | 1.45E+05 | ND | 6.94E+10 | 5.06E+05 | 8.11E+04 |
| 33 | 3.06E+07 | 1.16E+05 | ND | 1.94E+05 | 2.12E+06 | 3.04E+06 |
| 34 | 1.45E+05 | 1.63E+05 | ND | ND | ND | ND |
| 35 | 5.32E+05 | 6.63E+04 | ND | 2.64E+10 | 4.25E+08 | 1.25E+08 |
| 36 | 9.48E+09 | 3.02E+05 | 2.75E+05 | 1.31E+05 | 4.85E+05 | ND |
