# Supplementary material for: Development of an infection via sugar meal (IVSM) technique to infect mosquitoes (Diptera: Culicidae) with arthropod-borne viruses
Source: PLoS Negl Trop Dis. 2026 Nov 2;20(11):e0014715. doi: 10.1371/journal.pntd.0014715 (PMC13750647; doi:10.1371/journal.pntd.0014715)
Supplement: Supplementary file 5 [file pntd.0014715.s005.docx]

| **Table A.** MAYV RNA copy number/mL in *Ae. albopictus* after a 1 hr IVSM at 7 and 10 dpi. | | | | | | |
| --- | --- | --- | --- | --- | --- | --- |
| **Sample** | **7 dpi** | | | **10 dpi** | | |
|  | BD | LW | SL | BD | LW | SL |
| 1 | 4.70E+08 | ND | ND | 3.67E+08 | 2.89E+05 | ND |
| 2 | 1.65E+08 | ND | ND | 3.59E+11 | 2.70E+10 | ND |
| 3 | ND | ND | ND | 5.66E+09 | 4.54E+05 | ND |
| 4 | 7.07E+08 | 3.08E+04 | ND | 4.90E+10 | 3.36E+09 | ND |
| 5 | 2.70E+08 | ND | ND | 1.79E+08 | ND | ND |
| 6 | ND | ND | ND | 3.12E+11 | 1.76E+10 | ND |
| 7 | 2.63E+08 | ND | ND | 1.50E+09 | 3.78E+07 | ND |
| 8 | 5.31E+05 | ND | ND | 1.61E+10 | 4.68E+08 | ND |
| 9 | ND | ND | ND | 2.37E+10 | 2.24E+09 | ND |
| 10 | 1.01E+08 | ND | ND | 4.33E+08 | 1.75E+05 | ND |
| 11 | 7.26E+06 | ND | ND | 2.31E+07 | ND | ND |
| 12 | 2.55E+08 | ND | ND | 2.10E+09 | 4.80E+05 | ND |
| 13 | 1.11E+05 | ND | ND | 3.58E+11 | 1.65E+10 | 1.52E+06 |
| 14 | 1.04E+09 | 4.13E+04 | ND | 2.90E+11 | 1.29E+10 | 2.82E+04 |
| 15 | 6.09E+09 | 1.78E+05 | ND | 2.29E+11 | 9.57E+09 | 2.08E+04 |
| 16 | 9.04E+08 | ND | ND | 2.34E+07 | 1.01E+07 | ND |
| 17 | 6.99E+08 | ND | ND | 2.60E+11 | 9.01E+09 | ND |
| 18 | 1.79E+09 | 1.15E+05 | ND | 2.51E+07 | 5.56E+05 | ND |
| 19 | 7.10E+07 | ND | ND | 3.61E+11 | 1.38E+10 | ND |
| 20 | 3.40E+07 | 2.67E+04 | ND | 5.01E+11 | 1.73E+10 | 2.36E+04 |
| 21 | 7.39E+05 | ND | ND | 2.27E+11 | 9.10E+09 | ND |
| 22 | ND | ND | ND | 2.11E+10 | 4.05E+09 | ND |
| 23 | 1.19E+08 | 2.54E+04 | ND | 2.76E+08 | 4.20E+05 | ND |
| 24 | 3.04E+07 | 3.11E+04 | ND | 6.83E+07 | 6.09E+05 | ND |
| 25 | 1.95E+07 | ND | ND | 9.77E+10 | 8.73E+09 | ND |
| 26 | 2.79E+09 | 4.05E+05 | ND | 5.00E+11 | 1.20E+10 | 7.63E+04 |
| 27 | 2.10E+06 | 6.54E+04 | 2.36E+05 | ND | ND | ND |
| 28 | 8.73E+07 | ND | ND | 1.01E+11 | 1.78E+10 | ND |
| 29 | 1.05E+07 | ND | ND | 1.71E+09 | 6.32E+05 | ND |
| 30 | - | - | - | 2.76E+08 | 2.60E+05 | ND |
| 31 | - | - | - | 6.20E+08 | 5.24E+05 | ND |
| 32 | - | - | - | 1.88E+11 | 7.77E+09 | 3.00E+06 |
| 33 | - | - | - | 9.60E+10 | 6.81E+09 | ND |
| 34 | - | - | - | 9.69E+08 | 6.87E+06 | ND |
| 35 | - | - | - | 9.37E+07 | 4.10E+05 | ND |
| 36 | - | - | - | 1.38E+09 | 3.22E+08 | 7.37E+04 |

| **Table B.** MAYV RNA copy number/mL in *Ae. albopictus* after a 24 hr IVSM at 7 and 10 dpi. | | | | | | |
| --- | --- | --- | --- | --- | --- | --- |
| **Sample** | **7 dpi** | | | **10 dpi** | | |
|  | BD | LW | SL | BD | LW | SL |
| 1 | 1.88E+06 | ND | ND | 2.17E+08 | ND | ND |
| 2 | 2.54E+06 | ND | ND | 5.87E+08 | 1.22E+06 | ND |
| 3 | 9.37E+10 | 2.57E+09 | ND | 1.10E+08 | ND | ND |
| 4 | 6.62E+08 | 9.55E+04 | ND | 2.81E+06 | 5.03E+05 | ND |
| 5 | 2.59E+07 | 3.76E+04 | ND | 2.06E+10 | 2.89E+08 | ND |
| 6 | 1.57E+08 | ND | ND | 6.31E+07 | ND | ND |
| 7 | 3.19E+05 | 2.75E+04 | ND | 3.78E+09 | ND | ND |
| 8 | 1.58E+05 | 5.48E+04 | ND | 7.30E+05 | 2.68E+05 | ND |
| 9 | 6.38E+08 | 4.35E+04 | ND | 3.84E+11 | 7.12E+09 | ND |
| 10 | 3.97E+11 | 8.79E+09 | ND | 6.84E+10 | 6.27E+07 | ND |
| 11 | 5.52E+07 | 1.36E+05 | ND | 6.49E+06 | ND | ND |
| 12 | 1.06E+12 | 4.75E+09 | ND | 1.64E+07 | ND | ND |
| 13 | 2.37E+08 | 1.09E+05 | ND | 1.03E+11 | 9.84E+09 | ND |
| 14 | 8.26E+08 | 1.00E+05 | ND | 5.03E+07 | 1.13E+06 | ND |
| 15 | 1.81E+08 | ND | ND | 1.87E+10 | 8.87E+08 | 2.15E+05 |
| 16 | 5.56E+07 | 2.16E+04 | 1.52E+04 | 1.35E+10 | 1.16E+08 | ND |
| 17 | 1.20E+05 | 5.18E+04 | ND | 2.77E+09 | 1.17E+07 | ND |
| 18 | 5.42E+11 | 1.28E+10 | ND | 7.18E+11 | 1.06E+10 | 2.99E+05 |
| 19 | 3.40E+09 | 1.55E+09 | ND | 9.72E+10 | 5.80E+09 | ND |
| 20 | ND | ND | ND | 5.63E+07 | 1.71E+06 | ND |
| 21 | 7.29E+08 | ND | ND | 5.85E+09 | 6.60E+06 | ND |
| 22 | 6.04E+06 | 7.09E+04 | ND | 1.70E+10 | 4.49E+08 | ND |
| 23 | 1.15E+05 | 2.35E+05 | ND | 1.06E+08 | 5.47E+05 | ND |
| 24 | 1.07E+08 | 2.60E+04 | ND | 1.31E+11 | 6.52E+09 | ND |
| 25 | 5.91E+08 | 5.91E+04 | ND | 1.94E+10 | 4.59E+06 | ND |
| 26 | 2.90E+09 | 2.87E+06 | ND | 1.51E+07 | 6.80E+05 | ND |
| 27 | 9.83E+07 | 5.22E+04 | ND | 3.39E+11 | 5.29E+09 | ND |
| 28 | 9.79E+10 | 1.49E+09 | ND | 2.18E+07 | 4.66E+06 | ND |
| 29 | 9.71E+10 | 2.21E+09 | ND | 2.20E+07 | 4.87E+05 | ND |
| 30 | 4.77E+09 | 1.18E+06 | ND | 7.03E+07 | 7.79E+05 | ND |
| 31 | 3.88E+06 | 7.47E+04 | ND | 1.04E+12 | 3.25E+09 | ND |
| 32 | - | - | - | 2.47E+08 | 1.70E+06 | ND |
| 33 | - | - | - | 7.33E+08 | 4.31E+05 | ND |

| **Table C.** MAYV RNA copy number/mL in *Cx. quinquefasciatus* after a 1 hr IVSM at 7 and 10 dpi. | | | | | | |
| --- | --- | --- | --- | --- | --- | --- |
| **Sample** | **7 dpi** | | | **10 dpi** | | |
|  | BD | LW | SL | BD | LW | SL |
| 1 | ND | ND | ND | ND | ND | ND |
| 2 | ND | ND | ND | ND | ND | ND |
| 3 | ND | ND | ND | ND | ND | ND |
| 4 | ND | ND | ND | ND | ND | ND |
| 5 | ND | ND | ND | ND | ND | ND |
| 6 | ND | ND | ND | ND | ND | ND |
| 7 | ND | ND | ND | 1.08E+04 | 1.25E+04 | ND |
| 8 | ND | ND | ND | ND | ND | ND |
| 9 | ND | ND | ND | ND | ND | ND |
| 10 | ND | ND | ND | ND | ND | ND |
| 11 | ND | ND | ND | ND | ND | ND |
| 12 | 1.02E+04 | ND | ND | 1.45E+04 | ND | ND |
| 13 | 7.96E+05 | 2.81E+05 | ND | ND | ND | ND |
| 14 | ND | ND | ND | ND | ND | ND |
| 15 | ND | ND | ND | 1.88E+04 | 3.98E+04 | ND |
| 16 | ND | ND | ND | ND | ND | ND |
| 17 | 1.14E+04 | 4.66E+04 | ND | ND | ND | ND |
| 18 | ND | ND | ND | ND | ND | ND |
| 19 | 6.97E+06 | 3.63E+05 | ND | ND | ND | ND |
| 20 | ND | ND | ND | ND | ND | ND |
| 21 | ND | ND | ND | ND | ND | ND |
| 22 | ND | ND | ND | ND | ND | ND |
| 23 | ND | ND | ND | ND | ND | ND |
| 24 | ND | ND | ND | ND | ND | ND |
| 25 | ND | ND | ND | ND | ND | ND |
| 26 | ND | ND | ND | 1.47E+04 | ND | ND |
| 27 | ND | ND | ND | ND | ND | ND |
| 28 | 2.66E+04 | 1.19E+04 | ND | ND | ND | ND |
| 29 | ND | ND | ND | - | - | - |

| **Table D.** MAYV RNA copy number/mL in *Cx. quinquefasciatus* after a 24 hr IVSM at 7 and 10 dpi. | | | | | | |
| --- | --- | --- | --- | --- | --- | --- |
| **Sample** | **7 dpi** | | | **10 dpi** | | |
|  | BD | LW | SL | BD | LW | SL |
| 1 | ND | ND | ND | ND | ND | ND |
| 2 | ND | ND | ND | ND | ND | ND |
| 3 | ND | ND | ND | ND | ND | ND |
| 4 | ND | ND | ND | ND | ND | ND |
| 5 | ND | ND | ND | ND | ND | ND |
| 6 | ND | ND | ND | ND | ND | ND |
| 7 | ND | ND | ND | ND | ND | ND |
| 8 | ND | ND | ND | ND | ND | ND |
| 9 | 1.16E+09 | 7.32E+07 | ND | ND | ND | ND |
| 10 | ND | ND | ND | ND | ND | ND |
| 11 | ND | ND | ND | ND | ND | ND |
| 12 | ND | ND | ND | ND | ND | ND |
| 13 | ND | ND | ND | ND | ND | ND |
| 14 | ND | ND | ND | ND | ND | ND |
| 15 | ND | ND | ND | ND | ND | ND |
| 16 | ND | ND | ND | ND | ND | ND |
| 17 | ND | ND | ND | ND | ND | ND |
| 18 | ND | ND | ND | ND | ND | ND |
| 19 | ND | ND | ND | ND | ND | ND |
| 20 | 1.87E+08 | 3.02E+08 | 2.54E+05 | ND | ND | ND |
| 21 | ND | ND | ND | ND | ND | ND |
| 22 | ND | ND | ND | ND | ND | ND |
| 23 | ND | ND | ND | ND | ND | ND |
| 24 | 3.08E+04 | ND | ND | ND | ND | ND |
| 25 | ND | ND | ND | ND | ND | ND |
| 26 | ND | ND | ND | ND | ND | ND |
| 27 | ND | ND | ND | ND | ND | ND |
| 28 | ND | ND | ND | ND | ND | ND |
| 29 | - | - | - | ND | ND | ND |
