# Supplementary material for: Development of an infection via sugar meal (IVSM) technique to infect mosquitoes (Diptera: Culicidae) with arthropod-borne viruses
Source: PLoS Negl Trop Dis. 2026 Nov 2;20(11):e0014715. doi: 10.1371/journal.pntd.0014715 (PMC13750647; doi:10.1371/journal.pntd.0014715)
Supplement: Supplementary file 6 [file pntd.0014715.s006.docx]

| **Table A.** ZIKV (PRVABC5) RNA copy number/mL in *Ae. albopictus* after a 1 hr IVSM at 7 and 10 dpi. | | | | | | |
| --- | --- | --- | --- | --- | --- | --- |
| **Sample** | **7 dpi** | | | **10 dpi** | | |
|  | BD | LW | SL | BD | LW | SL |
| 1 | ND | ND | ND | ND | ND | ND |
| 2 | ND | ND | ND | ND | ND | ND |
| 3 | ND | ND | ND | ND | ND | ND |
| 4 | ND | ND | ND | ND | ND | ND |
| 5 | ND | ND | ND | ND | ND | ND |
| 6 | ND | ND | ND | ND | ND | ND |
| 7 | 7.57E+07 | ND | ND | ND | ND | ND |
| 8 | ND | ND | ND | ND | ND | ND |
| 9 | ND | ND | ND | ND | ND | ND |
| 10 | ND | ND | ND | ND | ND | ND |
| 11 | ND | ND | ND | ND | ND | ND |
| 12 | ND | ND | ND | ND | ND | ND |
| 13 | ND | ND | ND | ND | ND | ND |
| 14 | ND | ND | ND | ND | ND | ND |
| 15 | ND | ND | ND | ND | ND | ND |
| 16 | ND | ND | ND | ND | ND | ND |
| 17 | ND | ND | ND | ND | ND | ND |
| 18 | ND | ND | ND | ND | ND | ND |
| 19 | ND | ND | ND | ND | ND | ND |
| 20 | ND | ND | ND | 1.07E+04 | ND | ND |
| 21 | ND | ND | ND | 3.65E+08 | 6.42E+06 | ND |
| 22 | ND | ND | ND | ND | ND | ND |
| 23 | ND | ND | ND | ND | ND | ND |
| 24 | 1.02E+09 | 4.30E+07 | ND | ND | ND | ND |
| 25 | 1.23E+08 | 2.00E+07 | ND | ND | ND | ND |
| 26 | ND | ND | ND | ND | ND | ND |
| 27 | ND | ND | ND | ND | ND | ND |
| 28 | 1.10E+07 | ND | ND | ND | ND | ND |
| 29 | ND | ND | ND | - | - | - |
| 30 | ND | ND | ND | - | - | - |
| 31 | ND | ND | ND | - | - | - |

| **Table B.** ZIKV (PRVABC5) RNA copy number/mL in *Ae. albopictus* after a 24 hr IVSM at 7 and 10 dpi. | | | | | | |
| --- | --- | --- | --- | --- | --- | --- |
| **Sample** | **7 dpi** | | | **10 dpi** | | |
|  | BD | LW | SL | BD | LW | SL |
| 1 | ND | ND | ND | ND | ND | ND |
| 2 | ND | ND | ND | ND | ND | ND |
| 3 | ND | ND | ND | ND | ND | ND |
| 4 | ND | ND | ND | ND | ND | ND |
| 5 | ND | ND | ND | ND | ND | ND |
| 6 | ND | ND | ND | ND | ND | ND |
| 7 | ND | ND | ND | ND | ND | ND |
| 8 | ND | ND | ND | ND | ND | ND |
| 9 | ND | ND | ND | ND | ND | ND |
| 10 | ND | ND | ND | ND | ND | ND |
| 11 | 1.84E+08 | 4.74E+07 | ND | ND | ND | ND |
| 12 | ND | ND | ND | ND | ND | ND |
| 13 | ND | ND | ND | ND | ND | ND |
| 14 | ND | ND | ND | 1.37E+04 | ND | ND |
| 15 | ND | ND | ND | ND | ND | ND |
| 16 | ND | ND | ND | ND | ND | ND |
| 17 | ND | ND | ND | ND | ND | ND |
| 18 | 2.48E+05 | ND | ND | ND | ND | ND |
| 19 | ND | ND | ND | ND | ND | ND |
| 20 | ND | ND | ND | 2.96E+03 | ND | ND |
| 21 | ND | ND | ND | ND | ND | ND |
| 22 | ND | ND | ND | 2.28E+07 | ND | ND |
| 23 | ND | ND | ND | ND | ND | ND |
| 24 | ND | ND | ND | ND | ND | ND |
| 25 | ND | ND | ND | ND | ND | ND |
| 26 | ND | ND | ND | 2.24E+03 | ND | ND |
| 27 | ND | ND | ND | ND | ND | ND |
| 28 | ND | ND | ND | ND | ND | ND |
| 29 | ND | ND | ND | ND | ND | ND |
| 30 | ND | ND | ND | - | - | - |
| 31 | ND | ND | ND | - | - | - |
| 32 | ND | ND | ND | - | - | - |
| 33 | ND | ND | ND | - | - | - |
| 34 | ND | ND | ND | - | - | - |

| **Table C.** ZIKV (R116265) RNA copy number/mL in *Ae. albopictus* after a 1 hr IVSM at 7 and 10 dpi. | | | | | | |
| --- | --- | --- | --- | --- | --- | --- |
| **Sample** | **7 dpi** | | | **10 dpi** | | |
|  | BD | LW | SL | BD | LW | SL |
| 1 | ND | ND | ND | ND | ND | ND |
| 2 | ND | ND | ND | ND | ND | ND |
| 3 | ND | ND | ND | ND | ND | ND |
| 4 | ND | ND | ND | ND | ND | ND |
| 5 | ND | ND | ND | ND | ND | ND |
| 6 | ND | ND | ND | ND | ND | ND |
| 7 | ND | ND | ND | ND | ND | ND |
| 8 | ND | ND | ND | ND | ND | ND |
| 9 | ND | ND | ND | ND | ND | ND |
| 10 | ND | ND | ND | ND | ND | ND |
| 11 | ND | ND | ND | ND | ND | ND |
| 12 | ND | ND | ND | ND | ND | ND |
| 13 | ND | ND | ND | ND | ND | ND |
| 14 | ND | ND | ND | ND | ND | ND |
| 15 | ND | ND | ND | 6.80E+08 | 1.44E+08 | ND |
| 16 | ND | ND | ND | ND | ND | ND |
| 17 | ND | ND | ND | ND | ND | ND |
| 18 | ND | ND | ND | ND | ND | ND |
| 19 | ND | ND | ND | ND | ND | ND |
| 20 | ND | ND | ND | ND | ND | ND |
| 21 | ND | ND | ND | ND | ND | ND |
| 22 | ND | ND | ND | ND | ND | ND |
| 23 | ND | ND | ND | ND | ND | ND |
| 24 | ND | ND | ND | ND | ND | ND |
| 25 | ND | ND | ND | ND | ND | ND |
| 26 | ND | ND | ND | ND | ND | ND |
| 27 | ND | ND | ND | ND | ND | ND |
| 28 | ND | ND | ND | ND | ND | ND |

| **Table D.** ZIKV (PRVABC5) RNA copy number/mL in *Cx. quinquefasciatus* after a 1 hr IVSM at 7 and 10 dpi. | | | | | | |
| --- | --- | --- | --- | --- | --- | --- |
| **Sample** | **7 dpi** | | | **10 dpi** | | |
|  | BD | LW | SL | BD | LW | SL |
| 1 | ND | ND | ND | ND | ND | ND |
| 2 | ND | ND | ND | ND | ND | ND |
| 3 | ND | ND | ND | ND | ND | ND |
| 4 | ND | ND | ND | ND | ND | ND |
| 5 | ND | ND | ND | ND | ND | ND |
| 6 | ND | ND | ND | ND | ND | ND |
| 7 | ND | ND | ND | ND | ND | ND |
| 8 | ND | ND | ND | ND | ND | ND |
| 9 | ND | ND | ND | ND | ND | ND |
| 10 | ND | ND | ND | 1.23E+04 | ND | ND |
| 11 | ND | ND | ND | ND | ND | ND |
| 12 | ND | ND | ND | ND | ND | ND |
| 13 | ND | ND | ND | ND | ND | ND |
| 14 | ND | ND | ND | ND | ND | ND |
| 15 | ND | ND | ND | ND | ND | ND |
| 16 | ND | ND | ND | ND | ND | ND |
| 17 | ND | ND | ND | ND | ND | ND |
| 18 | ND | ND | ND | ND | ND | ND |
| 19 | ND | ND | ND | ND | ND | ND |
| 20 | ND | ND | ND | ND | ND | ND |
| 21 | ND | ND | ND | ND | ND | ND |
| 22 | ND | ND | ND | ND | ND | ND |
| 23 | ND | ND | ND | ND | ND | ND |
| 24 | ND | ND | ND | ND | ND | ND |
| 25 | ND | ND | ND | ND | ND | ND |
| 26 | ND | ND | ND | ND | ND | ND |
| 27 | ND | ND | ND | ND | ND | ND |
| 28 | ND | ND | ND | ND | ND | ND |
| 29 | ND | ND | ND | ND | ND | ND |
| 30 | ND | ND | ND | ND | ND | ND |
| 31 | ND | ND | ND | ND | ND | ND |
| 32 | ND | ND | ND | ND | ND | ND |
| 33 | ND | ND | ND | ND | ND | ND |
| 34 | ND | ND | ND | ND | ND | ND |
| 35 | - | - | - | ND | ND | ND |

| **Table E.** ZIKV (PRVABC5) RNA copy number/mL in *Cx. quinquefasciatus* after a 24 hr IVSM at 7 and 10 dpi. | | | | | | |
| --- | --- | --- | --- | --- | --- | --- |
| **Sample** | **7 dpi** | | | **10 dpi** | | |
|  | BD | LW | SL | BD | LW | SL |
| 1 | ND | ND | ND | ND | ND | ND |
| 2 | ND | ND | ND | ND | ND | ND |
| 3 | ND | ND | ND | 2.99E+04 | 2.12E+04 | ND |
| 4 | ND | ND | ND | ND | ND | ND |
| 5 | ND | ND | ND | ND | ND | ND |
| 6 | 8.69E+05 | ND | ND | ND | ND | ND |
| 7 | 4.27E+05 | ND | ND | ND | ND | ND |
| 8 | ND | ND | ND | ND | ND | ND |
| 9 | ND | ND | ND | ND | ND | ND |
| 10 | ND | ND | ND | ND | ND | ND |
| 11 | ND | ND | ND | ND | ND | ND |
| 12 | 1.03E+05 | ND | ND | ND | ND | ND |
| 13 | ND | ND | ND | ND | ND | ND |
| 14 | ND | ND | ND | ND | ND | ND |
| 15 | ND | ND | ND | 3.93E+04 | ND | ND |
| 16 | ND | ND | ND | ND | ND | ND |
| 17 | ND | ND | ND | ND | ND | ND |
| 18 | ND | ND | ND | ND | ND | ND |
| 19 | ND | ND | ND | ND | ND | ND |
| 20 | ND | ND | ND | ND | ND | ND |
| 21 | ND | ND | ND | ND | ND | ND |
| 22 | ND | ND | ND | ND | ND | ND |
| 23 | ND | ND | ND | ND | ND | ND |
| 24 | ND | ND | ND | ND | ND | ND |
| 25 | ND | ND | ND | ND | ND | ND |
| 26 | ND | ND | ND | ND | ND | ND |
| 27 | 1.67E+06 | ND | ND | ND | ND | ND |
| 28 | ND | ND | ND | ND | ND | ND |
| 29 | ND | ND | ND | ND | ND | ND |
| 30 | ND | ND | ND | ND | ND | ND |
| 31 | - | - | - | ND | ND | ND |
| 32 | - | - | - | ND | ND | ND |
