# Supplementary material for: Development of an infection via sugar meal (IVSM) technique to infect mosquitoes (Diptera: Culicidae) with arthropod-borne viruses
Source: PLoS Negl Trop Dis. 2026 Nov 2;20(11):e0014715. doi: 10.1371/journal.pntd.0014715 (PMC13750647; doi:10.1371/journal.pntd.0014715)
Supplement: Supplementary file 7 [file pntd.0014715.s007.docx]

| **Table A.** WNV RNA copy number/mL in *Ae. albopictus* (2 and 7 dpe) after a 1 hr IVSM at 10 dpi. | | | | | | |
| --- | --- | --- | --- | --- | --- | --- |
| **Sample** | **2 dpe** | | | **7 dpe** | | |
|  | BD | LW | SL | BD | LW | SL |
| 1 | 1.59E+10 | 9.66E+08 | ND | 6.00E+05 | 3.28E+06 | ND |
| 2 | 2.34E+05 | 5.08E+05 | ND | 2.29E+09 | 1.42E+06 | ND |
| 3 | 1.25E+11 | 3.38E+08 | ND | 1.46E+12 | 2.92E+10 | 2.08E+09 |
| 4 | 1.13E+11 | 1.48E+09 | ND | 9.33E+11 | 9.87E+10 | 3.09E+08 |
| 5 | ND | ND | ND | 2.53E+07 | 8.37E+05 | ND |
| 6 | 4.58E+08 | 2.23E+05 | ND | 5.95E+11 | 1.83E+10 | 3.36E+05 |
| 7 | 7.80E+09 | 3.06E+05 | ND | 9.19E+06 | 1.12E+06 | ND |
| 8 | 1.61E+11 | 2.58E+09 | ND | ND | ND | ND |
| 9 | 5.27E+11 | 1.62E+10 | 1.29E+07 | 1.82E+11 | 2.55E+09 | ND |
| 10 | 8.58E+05 | 2.73E+05 | ND | 1.21E+06 | 2.56E+06 | ND |
| 11 | 6.42E+11 | 1.06E+10 | 1.40E+05 | 1.37E+12 | 2.25E+10 | 8.74E+07 |
| 12 | 8.75E+05 | 5.35E+05 | ND | 3.01E+04 | 8.27E+05 | ND |
| 13 | 4.32E+08 | 3.78E+04 | ND | 3.25E+11 | 1.31E+10 | ND |
| 14 | 4.91E+11 | 1.64E+10 | ND | ND | ND | ND |
| 15 | 5.71E+08 | 2.13E+06 | ND | 5.79E+04 | 2.45E+05 | ND |
| 16 | 1.45E+11 | 1.67E+10 | 2.99E+06 | 7.60E+12 | 1.14E+11 | 4.23E+08 |
| 17 | 3.32E+12 | 9.53E+10 | 2.93E+05 | 9.22E+12 | 2.92E+11 | ND |
| 18 | 2.03E+06 | 3.12E+04 | ND | 2.30E+10 | 3.04E+06 | ND |
| 19 | 3.94E+08 | 1.83E+05 | 1.37E+05 | 1.44E+11 | 4.57E+09 | ND |
| 20 | 1.47E+09 | 1.45E+05 | ND | - | - | - |
| 21 | ND | ND | ND | - | - | - |
| 22 | 2.09E+12 | 1.21E+11 | 1.44E+08 | - | - | - |

| **Table B.** WNV RNA copy number/mL in *Ae. albopictus* (2 and 7 dpe) after a 1 hr blood feeding at 10 dpi. | | | | | | |
| --- | --- | --- | --- | --- | --- | --- |
| **Sample** | **2 dpe** | | | **7 dpe** | | |
|  | BD | LW | SL | BD | LW | SL |
| 1 | ND | ND | ND | 2.67E+11 | 4.55E+09 | ND |
| 2 | 2.68E+11 | 1.87E+08 | ND | 3.26E+05 | 1.46E+05 | ND |
| 3 | 1.29E+09 | ND | ND | 2.20E+05 | ND | ND |
| 4 | 3.09E+10 | 5.22E+08 | 1.97E+05 | 7.31E+11 | 8.32E+09 | 5.78E+07 |
| 5 | 8.59E+08 | 9.99E+05 | ND | 6.41E+05 | ND | ND |
| 6 | ND | ND | ND | 6.06E+08 | 6.27E+04 | ND |
| 7 | 3.38E+11 | 1.99E+10 | 1.60E+07 | ND | ND | ND |
| 8 | 3.03E+06 | 1.44E+07 | ND | 7.57E+08 | 1.19E+06 | ND |
| 9 | 3.08E+10 | ND | ND | 5.37E+05 | 6.09E+04 | ND |
| 10 | 3.08E+05 | 1.13E+05 | ND | 4.82E+11 | 4.54E+08 | 2.70E+06 |
| 11 | 1.51E+05 | 1.12E+05 | ND | 3.70E+05 | ND | ND |
| 12 | 3.47E+12 | 8.33E+10 | 6.50E+06 | 1.28E+06 | ND | ND |
| 13 | 3.74E+05 | ND | ND | 6.06E+10 | 8.53E+08 | ND |
| 14 | ND | ND | ND | 7.10E+05 | 4.17E+05 | ND |
| 15 | 7.40E+12 | 2.75E+11 | 2.65E+07 | 4.88E+05 | 8.94E+04 | ND |
| 16 | 9.33E+11 | 9.94E+09 | 1.29E+07 | 5.36E+04 | 8.60E+05 | ND |
| 17 | 4.37E+10 | 1.91E+07 | ND | 1.40E+12 | 4.66E+10 | 1.79E+08 |
| 18 | ND | ND | ND | 2.90E+08 | 6.09E+06 | ND |
| 19 | 3.70E+09 | 4.62E+08 | ND | 5.84E+08 | 8.43E+07 | ND |
| 20 | - | - | - | 1.99E+12 | 3.84E+10 | 3.13E+09 |

| **Table C.** WNV RNA copy number/mL in *Cx. quinquefasciatus* (2 and 7 dpe) after a 1 hr IVSM at 10 dpi. | | | | | | |
| --- | --- | --- | --- | --- | --- | --- |
| **Sample** | **2 dpe** | | | **7 dpe** | | |
|  | BD | LW | SL | BD | LW | SL |
| 1 | 5.48E+10 | 1.80E+08 | 3.23E+06 | 6.34E+10 | 5.27E+09 | 1.07E+07 |
| 2 | 4.13E+07 | 1.41E+05 | ND | ND | ND | ND |
| 3 | 3.55E+07 | 4.13E+04 | ND | 2.49E+10 | 7.67E+06 | ND |
| 4 | 1.07E+07 | 1.39E+05 | ND | 3.44E+11 | 1.30E+10 | 4.95E+06 |
| 5 | ND | ND | ND | 7.29E+10 | 3.19E+09 | ND |
| 6 | 6.38E+10 | 4.35E+04 | ND | 1.75E+10 | 1.73E+09 | ND |
| 7 | 2.95E+11 | 1.03E+10 | 5.63E+08 | ND | ND | ND |
| 8 | 4.62E+05 | 1.55E+06 | ND | 2.14E+11 | 1.35E+10 | 6.17E+07 |
| 9 | 2.05E+11 | 6.34E+09 | 4.09E+07 | 1.55E+05 | 2.42E+06 | ND |
| 10 | 5.95E+08 | 8.05E+04 | ND | 1.78E+08 | 1.58E+05 | ND |
| 11 | 7.57E+08 | 1.25E+05 | ND | 2.29E+08 | 1.19E+06 | 1.65E+06 |
| 12 | 7.24E+09 | 5.81E+04 | ND | 6.79E+04 | 5.16E+04 | ND |
| 13 | 7.98E+09 | 8.97E+08 | ND | 2.28E+11 | 2.55E+09 | 5.54E+07 |
| 14 | 1.49E+08 | 3.71E+05 | ND | 2.07E+04 | ND | ND |
| 15 | ND | ND | ND | 3.57E+07 | 1.34E+05 | ND |
| 16 | 9.71E+07 | 5.98E+05 | ND | 1.40E+09 | 1.97E+05 | ND |
| 17 | 1.38E+11 | 6.13E+09 | 1.96E+08 | 2.88E+11 | 1.35E+10 | 9.56E+07 |
| 18 | 9.53E+05 | 9.68E+05 | ND | 1.10E+11 | 4.58E+09 | 1.91E+06 |

| **Table D.** WNV RNA copy number/mL in *Cx. quinquefasciatus* (2 and 7 dpe) after a 1 hr blood feeding at 10 dpi. | | | | | | |
| --- | --- | --- | --- | --- | --- | --- |
| **Sample** | **2 dpe** | | | **7 dpe** | | |
|  | BD | LW | SL | BD | LW | SL |
| 1 | 2.65E+10 | 5.00E+05 | ND | 3.56E+07 | 8.54E+04 | ND |
| 2 | ND | ND | ND | 2.74E+05 | ND | ND |
| 3 | 7.39E+09 | 2.59E+07 | 8.66E+04 | 6.64E+11 | 1.80E+10 | 7.19E+06 |
| 4 | 3.07E+09 | 3.19E+04 | ND | ND | ND | ND |
| 5 | 1.15E+07 | 2.66E+05 | ND | 1.77E+12 | 6.74E+10 | 1.78E+09 |
| 6 | ND | ND | ND | 2.46E+10 | 3.03E+06 | 1.10E+06 |
| 7 | 4.45E+09 | 2.59E+04 | ND | 6.05E+08 | 7.34E+05 | ND |
| 8 | 4.05E+07 | 1.50E+05 | ND | 1.68E+09 | 5.86E+04 | ND |
| 9 | 7.79E+09 | 1.08E+07 | ND | 2.31E+05 | ND | ND |
| 10 | 4.48E+09 | 1.21E+08 | 6.07E+05 | ND | ND | ND |
| 11 | ND | ND | ND | 6.41E+09 | 1.24E+06 | ND |
| 12 | ND | ND | ND | 1.06E+11 | 4.89E+09 | 8.02E+05 |
| 13 | 3.11E+08 | 9.59E+05 | ND | 1.61E+05 | 4.79E+05 | ND |
| 14 | 1.10E+10 | 2.12E+07 | ND | 1.00E+05 | 2.47E+05 | ND |
| 15 | 1.78E+11 | 2.68E+09 | 1.54E+09 | 6.98E+09 | 3.63E+06 | ND |
| 16 | ND | ND | ND | 2.26E+08 | 6.19E+06 | ND |
| 17 | 6.59E+09 | 7.73E+06 | 3.96E+05 | 2.66E+10 | 8.23E+06 | 1.47E+05 |
| 18 | 9.58E+09 | ND | ND | 3.55E+08 | 1.41E+05 | ND |
| 19 | - | - | - | ND | ND | ND |
